# Evidence for functional selectivity in TUDC- and *nor*UDCA-induced signal transduction via α_5_β_1_ integrin towards choleresis

**DOI:** 10.1038/s41598-020-62326-y

**Published:** 2020-04-02

**Authors:** Michele Bonus, Annika Sommerfeld, Natalia Qvartskhava, Boris Görg, Beatrice Stefanie Ludwig, Horst Kessler, Holger Gohlke, Dieter Häussinger

**Affiliations:** 1 Institute for Pharmaceutical and Medicinal Chemistry, Heinrich Heine University Düsseldorf, Düsseldorf, Germany; 2 Clinic for Gastroenterology, Hepatology and Infectious Diseases, Heinrich Heine University Düsseldorf, Düsseldorf, Germany; 3 Institute for Advanced Study and Center for Integrated Protein Science, Department of Chemistry, Technische Universität München, Garching, Germany; 4 John von Neumann Institute for Computing (NIC), Jülich Supercomputing Centre (JSC), and Institute for Complex Systems - Structural Biochemistry (ICS-6), Forschungszentrum Jülich GmbH, Jülich, Germany

**Keywords:** Computational biophysics, Integrins, Hepatocytes

## Abstract

Functional selectivity is the ligand-specific activation of certain signal transduction pathways at a receptor and has been described for G protein-coupled receptors. However, it has not yet been described for ligands interacting with integrins without αI domain. Here, we show by molecular dynamics simulations that four side chain-modified derivatives of tauroursodeoxycholic acid (TUDC), an agonist of α_5_β_1_ integrin, differentially shift the conformational equilibrium of α_5_β_1_ integrin towards the active state, in line with the extent of β_1_ integrin activation from immunostaining. Unlike TUDC, 24-*nor*-ursodeoxycholic acid (*nor*UDCA)-induced β_1_ integrin activation triggered only transient activation of extracellular signal-regulated kinases and p38 mitogen-activated protein kinase and, consequently, only transient insertion of the bile acid transporter Bsep into the canalicular membrane, and did not involve activation of epidermal growth factor receptor. These results provide evidence that TUDC and *nor*UDCA exert a functional selectivity at α_5_β_1_ integrin and may provide a rationale for differential therapeutic use of UDCA and *nor*UDCA.

## Introduction

Functional selectivity is the ligand-specific activation of certain signal transduction pathways at a receptor that can signal through multiple pathways^1^. On the molecular level, a ligand likely achieves this type of differential activation by stabilizing only a specific subset of receptor conformations, in particular those that favor interactions with only a specific subset of downstream signaling molecules^1^. This phenomenon has so far been described in detail only for G protein-coupled receptors (GPCRs)^2^, but the observation that α_M_β_2_ integrins respond differently to fibrinogen- and CD40L-binding has led to the suggestion that this model could be extended to integrins with an αI domain^3,4^. However, the phenomenon has not yet been described for ligands interacting with integrins lacking an αI domain. Furthermore, a direct connection between differentially ligand-induced integrin conformations and differences in signal transduction pathways downstream of the integrin has not yet been established.

We recently identified the hydrophilic bile acid tauroursodeoxycholic acid (TUDC) as a potent agonist of an α_5_β_1_ integrin-mediated signaling pathway towards choleresis^5,8^. α_5_β_1_ integrin is the predominant integrin isoform in the liver and lacks an αI domain^6^. After uptake into hepatocytes through the Na^+^/taurocholate cotransporting polypeptide (Ntcp), TUDC directly activates intracellular α_5_β_1_ integrins, followed by an activating phosphorylation of mitogen-activated protein kinases (MAPK) Erk-1/-2 and p38^MAPK^ ^5^. These signaling events strongly resemble those initiated in response to hypoosmotic or insulin-induced hepatocyte swelling^7–9^. There, mechano/swelling-sensitive α_5_β_1_ integrins in the plasma membrane become activated and trigger FAK-/c-Src-/MAPK-dependent signaling towards choleresis with Bsep and Mrp2 insertion into the canalicular membrane^7,10,11^. Chemical modifications of the ursodeoxycholane scaffold in TUDC (Supplementary Fig. 1) either completely abolished activity on α_5_β_1_ integrin or led to a compound that inhibited the TUDC-induced β_1_ integrin activation (taurocholic acid (TC))^5^.

Here, we tested to what extent side chain-modified derivatives of TUDC (24-*nor*-ursodeoxycholic acid (*nor*UDCA), its taurine conjugate (T*nor*UDCA), glycoursodeoxycholic acid (GUDC), and unconjugated UDCA; Supplementary Fig. 1) can directly activate α_5_β_1_ integrins and whether the signaling events downstream of integrin activation differ from those triggered by TUDC. To probe for differences in ligand-induced conformational changes in integrin at the atomistic level, we performed all-atom molecular dynamics (MD) simulations of α_5_β_1_ integrin bound to TUDC, *nor*UDCA, T*nor*UDCA, GUDC, UDCA, or TC of, in total, 3.6 μs length. In parallel, we studied the extent to which *nor*UDCA-, T*nor*UDCA-, GUDC-, or UDCA stimulate the activation of β_1_ integrins during perfusion of rat liver and compared the signaling events downstream of *nor*UDCA-mediated integrin activation with TUDC-mediated integrin activation. Our results demonstrate that *nor*UDCA directly activates α_5_β_1_ integrins in hepatocytes and provide evidence that TUDC and *nor*UDCA exert a functional selectivity for certain signal transduction pathways in α_5_β_1_ integrin.

## Results

### In MD simulations norUDCA induces conformational changes in the α_5_β_1_ integrin headpiece that have been linked to integrin activation

**Figure 1:**
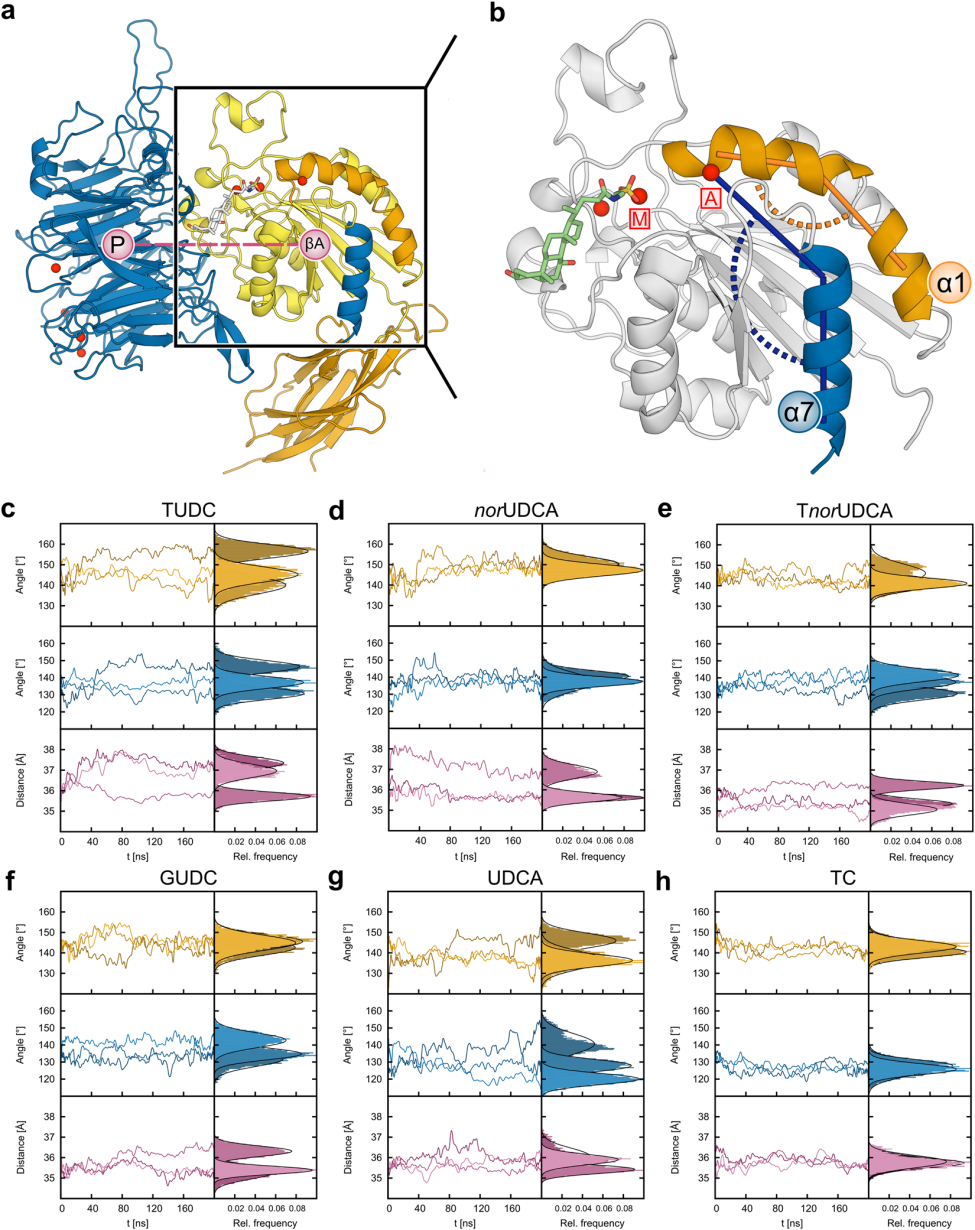
Conformational changes in the α_5_β_1_ integrin headpiece. (**a**) Part of the α_5_β_1_ integrin headpiece in cartoon representation. Helices α1 and α7 are highlighted in orange and blue. The propeller-βA distance is measured between the respective centers of mass (pink circles). Colors of the domains are according to Supplementary Fig. 19B. (**b**) Close-up view of the βA domain with the docked TUDC structure (stick representation)^5^. This complex structure was used to generate other starting structures by modifying the bile acid. Angles measured during the course of the MD simulations: orange: α1 kink angle; blue: α7 tilt angle. Mg^2+^ ions are depicted as red spheres; the one at the MIDAS site is labeled M, the one at the ADMIDAS A. (**c–h**) α1 kink angle (orange), α7 tilt angle (blue), and propeller-βA distance (pink) during the course of three (color shades) MD simulations of each of the complexes between α_5_β_1_ integrin and (**c**) TUDC, (**d**) *nor*UDCA, (**e**) T*nor*UDCA, (**f**) GUDC, (**g**) UDCA, and (**h**) TC. For clarity, the time course data (left) has been smoothed by Bezier curves. Relative frequencies of the parameters (right) are calculated for the last 100 ns of each simulation. The frequency distributions have been overlaid with Gaussians according to their means and standard deviations (black curves).

**Figure 2:**
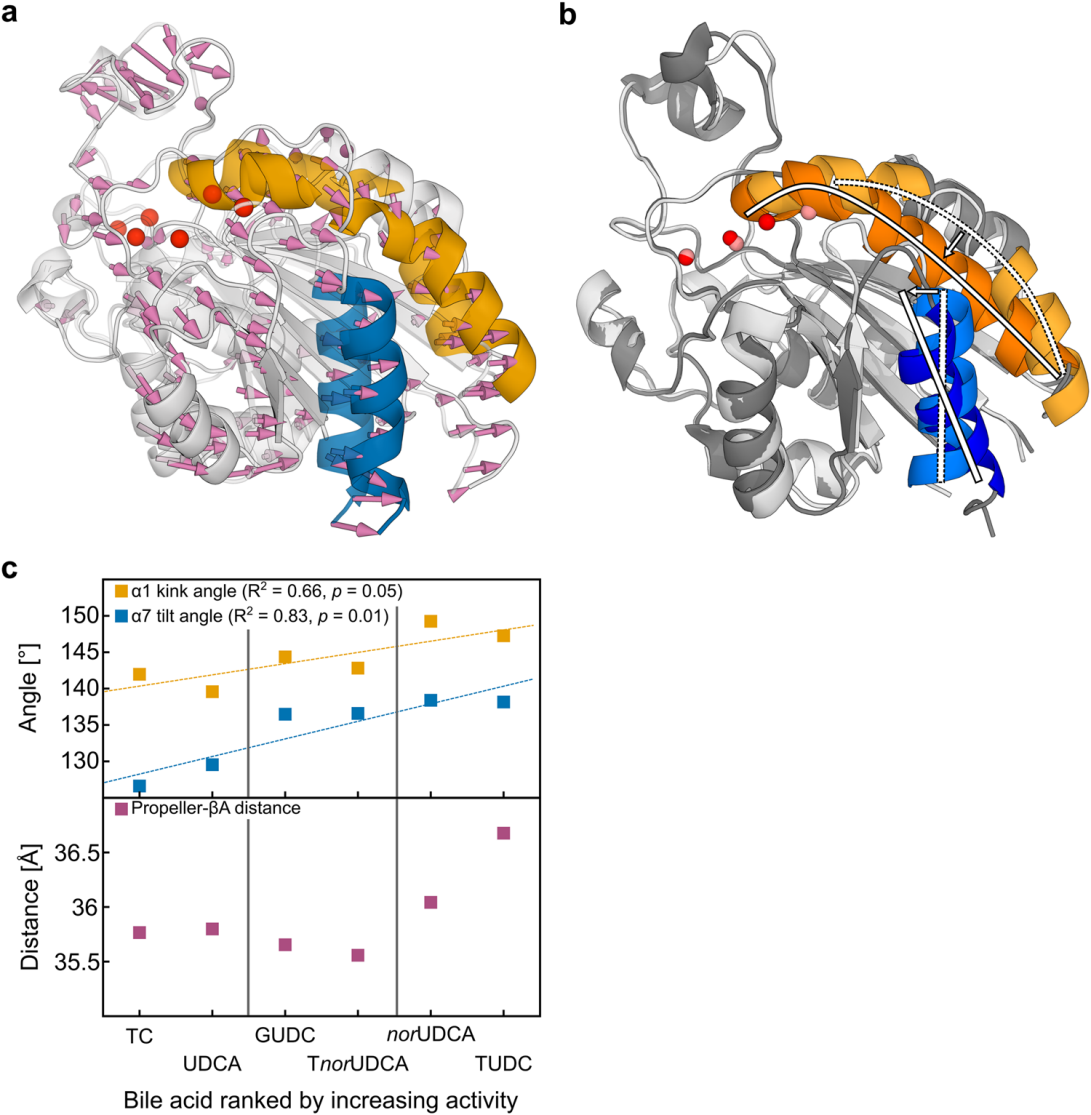
Activation of α_5_β_1_ integrins in MD simulations compared to activation of α_IIb_β_3_ integrin in crystal structures. (**a**) Structural overlay of the βA domain (transparent: starting structure; opaque: closest-to-average structure from the last 100 ns) by fitting on the β-propeller domain^5^. Pink arrows denote the positional shift of the βA domain relative to the β-propeller domain, resulting in an increased propeller-βA domain distance. (**b**) Overlay of the closed (lighter colors; PDB ID 3FCS) and open (darker colors; PDB ID 3FCU) conformations of the βA domain in α_IIb_β_3_ integrins. Straightening of the α1 helix (orange) and tilting of the α7 helix (blue) are indicated by white arcs and bars. (**c**) Average of the α1 kink angle (yellow), α7 tilt angle (blue), and β-propeller – βA-domain distance (magenta) over three replicates of MD simulations versus the rank of the bile acids according to their agonist activity towards α_5_β_1_ integrin as observed in Fig. 3A-D and ref. ^5^. Dashed lines represent correlation lines; fit parameters are given in the figures. Vertical lines separate the dataset into inactive (left), weakly active (middle), and highly active (right) bile acids.

We analyzed all-atom MD simulations of TUDC, *nor*UDCA, T*nor*UDCA, GUDC, UDCA, and TC bound to the shallow crevice in the subunit interface of the ectodomain of α_5_β_1_ integrin for conformational changes in the headpiece region that govern integrin activation. We described these conformational changes by means of three geometric parameters: the kink angle in helix α1, the α7 tilt angle, and the β-propeller – βA domain distance (Figs. 1, 2; details in the Methods subsection “*Analysis of MD trajectories*”). For each complex, three replicates were simulated for 200 ns length to probe for the statistical significance and convergence of the simulation results (Fig. 1c–h). All systems were stable over the course of the simulation time, as demonstrated by the time courses of the root-mean-square deviation (RMSD) of the atomic positions in the βA domain and the full protein (Supplementary Fig. 2), as well as the domain-wise minimum, maximum and average RMSD values (Supplementary Table 1). MD simulations of TUDC- and TC-bound ectodomains served as references as they display the occurrence or absence of conformational changes when the ectodomain is bound to an activating or inhibitory bile acid, respectively^5^. Particularly, TUDC leads to a kink angle of helix α1 of 147.3 ± 0.1°, a tilt angle of helix α7 of 138.2 ± 0.1°, and a distance between the βA-domain and the β-propeller of 36.68 ± 0.01 Å, whereas TC leads to angles of 142.0 ± 0.1° and 126.6 ± 0.1°, respectively, and a distance of 35.77 ± 0.01 Å (Figs. 1c,h and 2c, Supplementary Table 2). *nor*UDCA induces α1 kink and α7 tilt angles similar in magnitude as in the case of TUDC (149.2 ± 0.1° and 138.2 ± 0.1°, respectively), whereas the distance between β-propeller and βA-domain is ~0.6 Å smaller (Figs. 1d and 2a,c, Supplementary Table 2). These findings indicate that *nor*UDCA can activate α_5_β_1_ in a similar manner as TUDC but with a lower efficacy. In comparison with *nor*UDCA, for T*nor*UDCA- and GUDC-bound ectodomains, the α1 kink angle is decreased by ~6°, while the α7 tilt angle remains in the same range, being ~2° smaller. Furthermore, the β-propeller – βA domain distance is reduced by ~0.4 Å (Figs. 1e,f and 2c, Supplementary Table 2). Finally, for the UDCA-bound ectodomain, the α1 kink angle is 139.6 ± 0.1°, and the α7 tilt angle is 129.6 ± 0.1°; the β-propeller − βA domain distance is 35.80 ± 0.01 Å (Figs. 1g and 2c, Supplementary Table 2). These geometric parameters are more similar to those of TC than to those of any of the previously mentioned bile acids, indicating that UDCA, like TC^5^, cannot activate α_5_β_1_ integrins. Furthermore, by comparison, the above results for T*nor*UDCA- and GUDC indicate that the efficacy of these bile acids in activating α_5_β_1_ integrins is, at best, very weak. In all, the computational studies provide evidence that suggests that *nor*UDCA can directly activate α_5_β_1_ integrins.

### norUDCA activates β_1_ integrins in perfused rat liver

**Figure 3:**
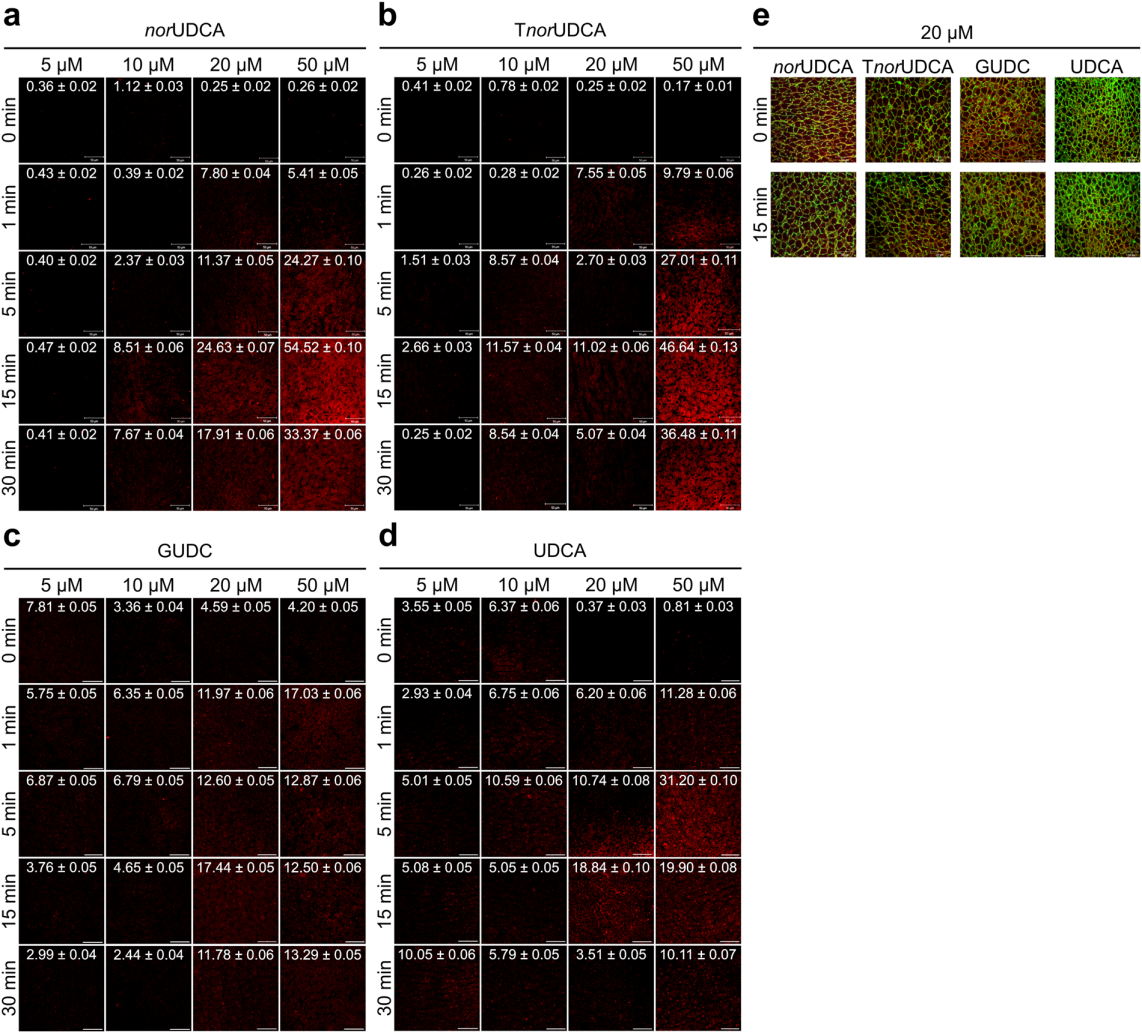
Effect of *nor*UDCA, T*nor*UDCA, GUDC, and UDCA on β_1_ integrin activation. Rat livers were perfused with (**a**) *nor*UDCA, (**b**) T*nor*UDCA, (**c**) GUDC, and (**d**) UDCA for up to 60 min with the concentrations indicated. Liver samples were immunostained for the active conformation of β_1_ integrin (red). The scale bar corresponds to 50 µm. Representative pictures of at least three independent experiments are depicted. To enhance visibility of the images, the white point of all channels in the RGB color space was reduced from the standard value of 255 to a value of 128. For each image, pixel intensities are indicated as average ± SEM. *Nor*UDCA and T*nor*UDCA triggered activation of the β_1_ integrin subunit within 15 min, with stronger effects observed with *nor*UDCA. In contrast, equimolar concentrations of UDCA and GUDC were ineffective. Like TUDC (Fig. 4)^5^, *nor*UDCA-induced β_1_ integrin activation occurred primarily in the intracellular compartment of hepatocytes. (**e**) Staining of total α_5_β_1_ integrin (red) and filamentous actin labeled with FITC-coupled phalloidin (green) at *t* = 0 min and *t* = 15 min after perfusion with *nor*UDCA, TUDC, GUDC, and UDCA.

In isolated perfused rat liver, addition of *nor*UDCA at a concentration of ≥ 20 µmol/l induces the appearance of the active conformation of β_1_ integrin after 15 min, whereas in the absence of *nor*UDCA, active β_1_ integrin was only scarcely detectable (Fig. 3a). As described for TUDC^5^, β_1_ integrin activation was predominantly observed inside the hepatocyte (Fig. 3a, Supplementary Fig. 3). In contrast, active β_1_ integrin was only weakly detectable with T*nor*UDCA (≥ 20 µmol/l) and GUDC ( ≥ 20 µmol/l) (Fig. 3b,c). Perfusion with UDCA ( ≥ 20 µmol/l) induces a stronger appearance of the active conformation of β_1_ integrin than do T*nor*UDCA and GUDC (Fig. 3d). None of the bile acids had any effect on the immunostaining for total α_5_β_1_ integrins (Fig. 3e).

### TUDC induces a stronger appearance of active β_1_ integrin than norUDCA

**Figure 4:**
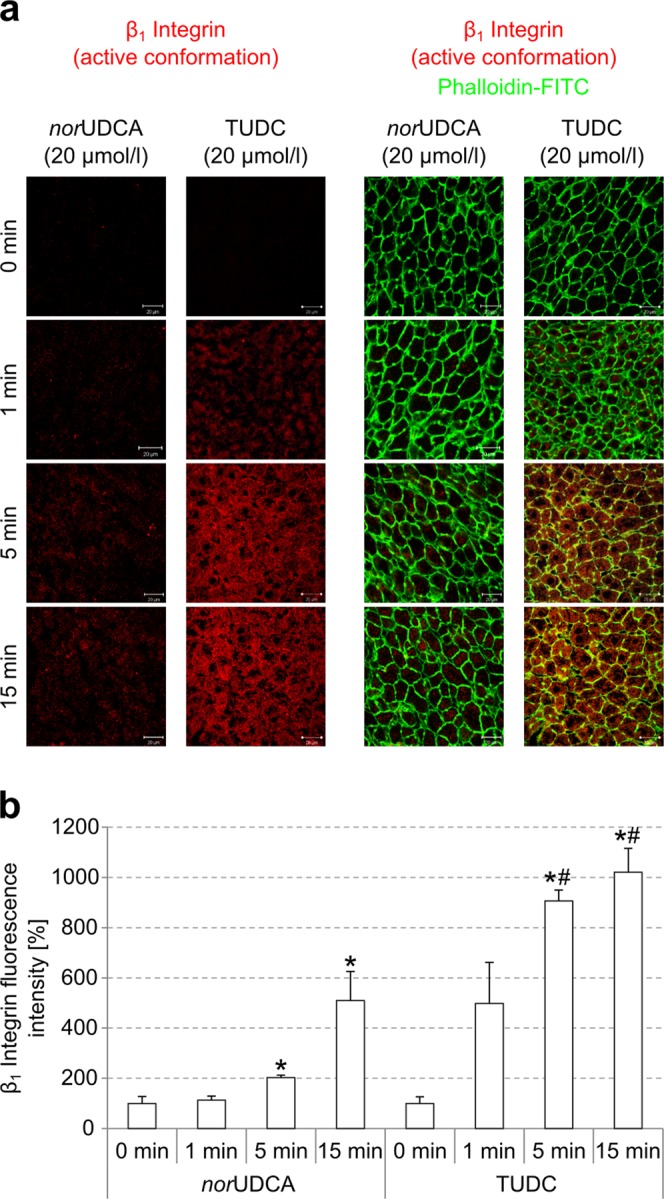
Immunofluorescence staining and quantification of β_1_ integrin. (**a**) Rat livers were perfused with either *nor*UDCA or TUDC (20 µmol/l each) for up to 15 min and immunostained for the active β_1_ integrin conformation and actin as given under “Experimental Procedures”. The scale bar corresponds to 20 µm. Representative pictures of three independent experiments are depicted. (**b**) β_1_ integrin fluorescence was quantified by using ImageJ analysis software. Whereas TUDC induced β_1_ integrin activation within 5 min, *nor*UDCA activated β_1_ integrins with lower effect. *p < 0.05 denotes statistical significance compared with the unstimulated control; #*p* < 0.05 statistical significance between *nor*UDCA and TUDC perfusion.

We compared the effect of *nor*UDCA at 20 µmol/l in inducing the appearance of the active conformation of β_1_ integrin in isolated perfused rat liver to that of TUDC at equimolar concentration as a known activator of α_5_β_1_ integrin (Fig. 4)^5^. Whereas TUDC induced a pronounced and significant β_1_ integrin activation within 5 min, as shown by a β_1_ integrin fluorescence intensity of 906 ± 43% relative to unstimulated control, *nor*UDCA activated β_1_ integrins with a lower effect (β_1_ integrin fluorescence intensity of 203 ± 10% relative to unstimulated control). After 5 and 15 min, β_1_ integrin fluorescence intensity was significantly higher in the presence of TUDC than in the presence of *nor*UDCA. After 15 min, *nor*UDCA-induced β_1_ integrin activation was 510% ± 116% of baseline (Fig. 4).

### norUDCA induces integrin-dependent signaling cascades similar to TUDC

**Figure 5:**
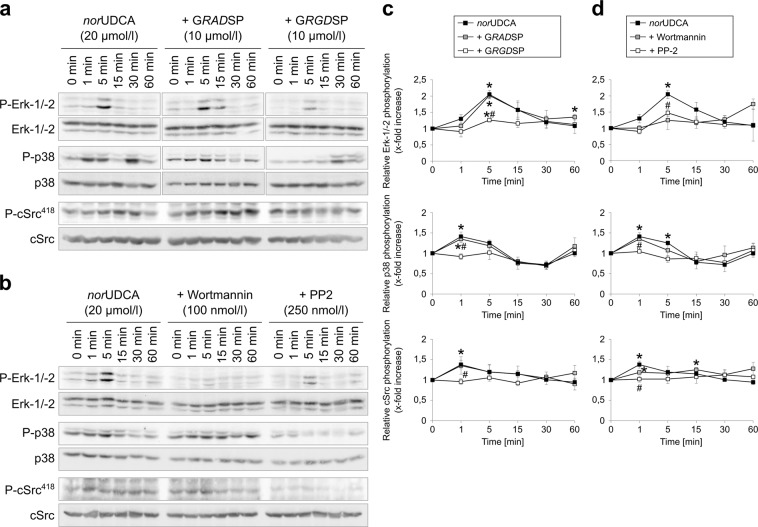
*nor*UDCA-induced activation of Erk-1/-2, p38^MAPK^ and Src. Rat livers were perfused with *nor*UDCA (20 µmol/l) for up to 60 min. Liver samples were taken at the time points indicated. The integrin antagonistic peptide (G*RGD*SP, 10 µmol/l), the inactive control peptide (G*RAD*SP, 10 µmol/l), the PI3-K inhibitor wortmannin (100 nmol/l), and the Src inhibitor PP-2 (250 nmol/l) were added 30 min prior to the addition of *nor*UDCA. Activation of Erk-1/-2, p38^MAPK^ and c-Src was analyzed by (**a,b**) Western blot using specific antibodies and (**c,d**) subsequent densitometric analysis. Total Erk-1/-2, total p38^MAPK^, and total c-Src served as respective loading control. Phosphorylation at *t* = 0 min was arbitrarily set as 1. Densitometric analyses (means ± SEM) and representative blots of at least three independent perfusion experiments are shown. *p < 0.05 statistical significance compared with the unstimulated control. #*p* < 0.05 statistical significance between *nor*UDCA in the absence and presence of an inhibitor. *nor*UDCA led to a significant activation of Erk-1/-2, p38 MAPK as well as c-Src in the perfused rat liver, which was inhibited by G*RGD*SP, whereas G*RAD*SP had no effect. Phosphorylation of Erk-1/-2, p38^MAPK^, and c-Src was sensitive to PP-2, whereas wortmannin inhibited Erk-1/2 and c-Src activation. Blots were cropped to focus on the area of interest, and full-length blots are presented in Supplementary Figs. 5 and 6.

**Figure 6:**
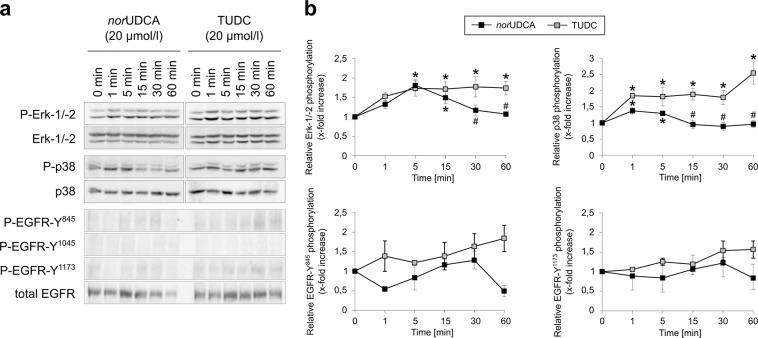
Comparison between *nor*UDCA- and TUDC-induced Erk-1/-2, p38^MAPK^ and EGFR activation. Rat livers were perfused with *nor*UDCA or TUDC (20 µmol/l each) for up to 60 min as described in “Experimental Procedures”. Liver samples were taken at the time points indicated. Phosphorylation of Erk-1/-2, p38^MAPK^, and EGFR tyrosine residues Tyr^845^, Tyr^1045^, and Tyr^1173^ was analyzed by (**a**) Western blot using specific antibodies and (**b**) subsequent densitometric analysis (black squares, *nor*UDCA; gray squares, TUDC). Total Erk-1/-2, total p38^MAPK^, and total EGFR served as respective loading controls. Phosphorylation at *t* = 0 was arbitrarily set to 1. Data represent the mean (mean ± SEM) of at least three independent experiments; *p < 0.05 statistical significance compared with the unstimulated control. #*p* < 0.05 statistical significance between *nor*UDCA and TUDC. Blots were cropped to focus on the area of interest, and full-length blots are presented in Supplementary Figure 10. TUDC led to activation of Erk-1/-2, p38^MAPK^, and EGFR, as indicated by phosphorylation of the EGFR tyrosine residues Tyr^845^ and Tyr^1173^. *nor*UDCA induced a transient Erk-1/-2 phosphorylation and a weak p38^MAPK^ activation. No EGFR activation was observed in *nor*UDCA-perfused livers.

Like TUDC^5^, *nor*UDCA (20 µmol/l) induced within 5 min phosphorylation of extracellular signal-regulated kinases Erk-1/-2, which was abolished in the presence of the RGD motif-containing hexapeptide GRGDSP (10 µmol/l) but not in the presence of the inactive control hexapeptide GRADSP (10 µmol/l) (Figs. 5 and 6). Erk-1/-2 phosphorylation due to *nor*UDCA did not increase when phosphatases were inhibited by okadaic acid (5 nmol/l), in contrast to TUDC-induced Erk-1/-2 phosphorylation (Supplementary Fig. 4). *nor*UDCA also increased activation of p38^MAPK^ and the activating Src phosphorylation at tyrosine 418 in an RGD hexapeptide-sensitive way (Fig. 5, Supplementary Fig. 5). PP-2 (250 nmol/l)^12^, an inhibitor of Src kinase, largely abolished the *nor*UDCA-induced Erk-1/-2 and p38^MAPK^ activation (Fig. 5, Supplementary Fig. 6). Thus, *nor*UDCA signaling to both Erk-1/-2 and p38^MAPK^ involves integrins and Src. In order to examine a possible involvement of PI3-K in *nor*UDCA-induced signaling, the specific inhibitor wortmannin (100 nmol/l) was preperfused. *nor*UDCA-induced activation of Erk-1/2 was largely suppressed when wortmannin was present (Fig. 5, Supplementary Fig. 6). In contrast, activation of Src and p38^MAPK^ was not inhibited by wortmannin (Fig. 5, Supplementary Fig. 6). These findings indicate that Src phosphorylation is upstream of PI3-K activation and that PI3-K is not involved in the signaling towards p38^MAPK^ activation. In control perfusion experiments without addition of *nor*UDCA, no effect on the phosphorylation of Erks, p38^MAPK^, or Src at tyrosine 418 was found (Supplementary Fig. 7). Next, we examined whether already the initial signaling pathways that usually follow integrin activation are differentially affected by TUDC or *nor*UDCA. Perfusion with TUDC (20 µmol/l) induced a significant integrin-mediated FAK^Y397^ autophosphorylation after 10 min (1.87 ± 0.24-fold amount of FAK^Y397-P^) that lasted for up to 30 min compared to livers perfused with normoosmotic medium (Supplementary Fig. 8). In contrast, perfusion with *nor*UDCA (20 µmol/l) led to an only transient FAK^Y397^ autophosphorylation that was maximal after 5 min (1.73 ± 0.35-fold amount of FAK^Y397-P^). Similar findings were obtained for other FAK phosphorylation sites, i.e. FAK^Y407^, FAK^Y576/577^, FAK^Y861^, and FAK^Y925^ (Supplementary Fig. 9).

### norUDCA does not induce epidermal growth factor receptor (EGFR)-dependent amplification of Erk-1/-2 and p38^MAPK^ signaling

**Figure 7:**
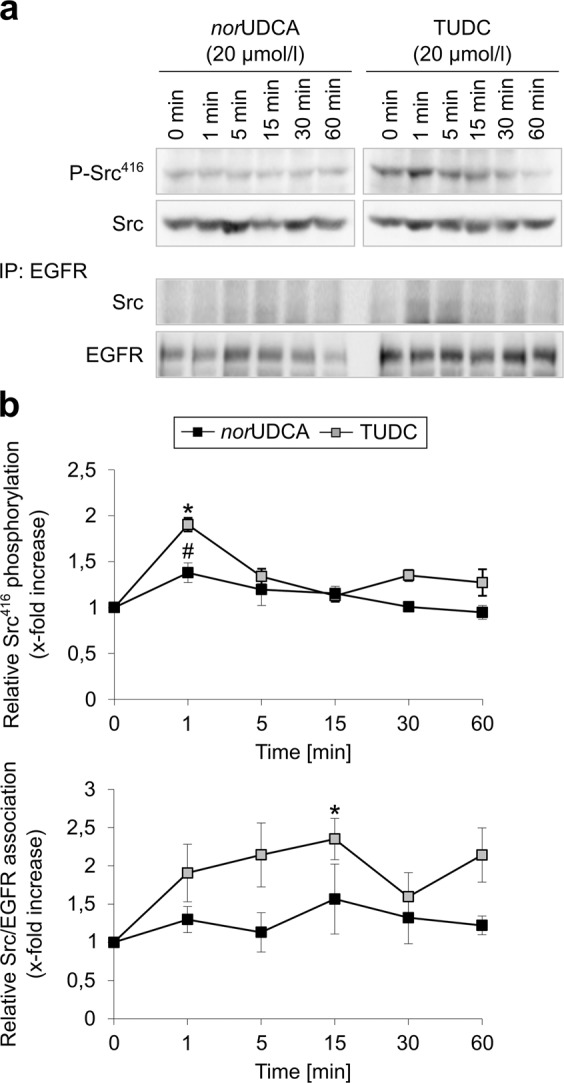
Comparison between *nor*UDCA- and TUDC-induced c-Src activation and EGFR/c-Src association. Rat livers were perfused with *nor*UDCA or TUDC (20 µmol/l each) for up to 60 min. Liver samples were taken at the time points indicated. Activation of c-Src was analyzed by (**a**) Western blot using specific antibodies and (**b**) subsequent densitometric analysis. Total c-Src served as respective loading control. EGFR was immunoprecipitated as described under “Experimental Procedures”. Samples were then analyzed for EGFR/c-Src association by detection of c-Src. Total EGFR served as a loading control. Phosphorylation at *t* = 0 min was set as 1. Densitometric analyses (means ± SEM) and representative blots of at least three independent perfusion experiments are shown. *p < 0.05 statistical significance compared with the unstimulated control. #*p* < 0.05 statistical significance between *nor*UDCA and TUDC. Blots were cropped to focus on the area of interest, and full-length blots are presented in Supplementary Figure 11. TUDC led to a significantly more intense phosphorylation of c-Src and EGFR/c-Src association than *nor*UDCA.

Dual activation of Erk-1/-2 and p38^MAPK^ is involved in the stimulation of canalicular secretion by TUDC. In contrast to TUDC, the effect of *nor*UDCA on Erk-1/-2 phosphorylation was transient and disappeared largely within 30 min of *nor*UDCA exposure (Fig. 6, Supplementary Fig. 10). After 15 min, *nor*UDCA-triggered p38^MAPK^ activation was also significantly lower than TUDC-induced phosphorylation of p38^MAPK^ (Fig. 6, Supplementary Fig. 10). Whereas TUDC induced phosphorylation of the EGFR tyrosine residues 845 and 1173, but not of Tyr1045 (Fig. 6, Supplementary Fig. 10), in line with previous data^5^, no activating phosphorylation of EGFR occurred in the presence of *nor*UDCA (Fig. 6, Supplementary Fig. 10). TUDC-induced EGFR trans-activation requires an EGFR/c-Src association following Src activation. Compared to the TUDC-triggered Src activation, the *nor*UDCA-induced activation of Src was significantly lower (Fig. 7, Supplementary Fig. 11). Immunoprecipitation studies of perfused liver samples revealed that EGFR/c-Src association was absent in *nor*UDCA-perfused livers (Fig. 7, Supplementary Fig. 11).

### TUDC-induced dual activation of Erk-1/-2 and p38^MAPK^, and Bsep insertion into the canalicular membrane, are dependent on EGFR phosphorylation

**Figure 8:**
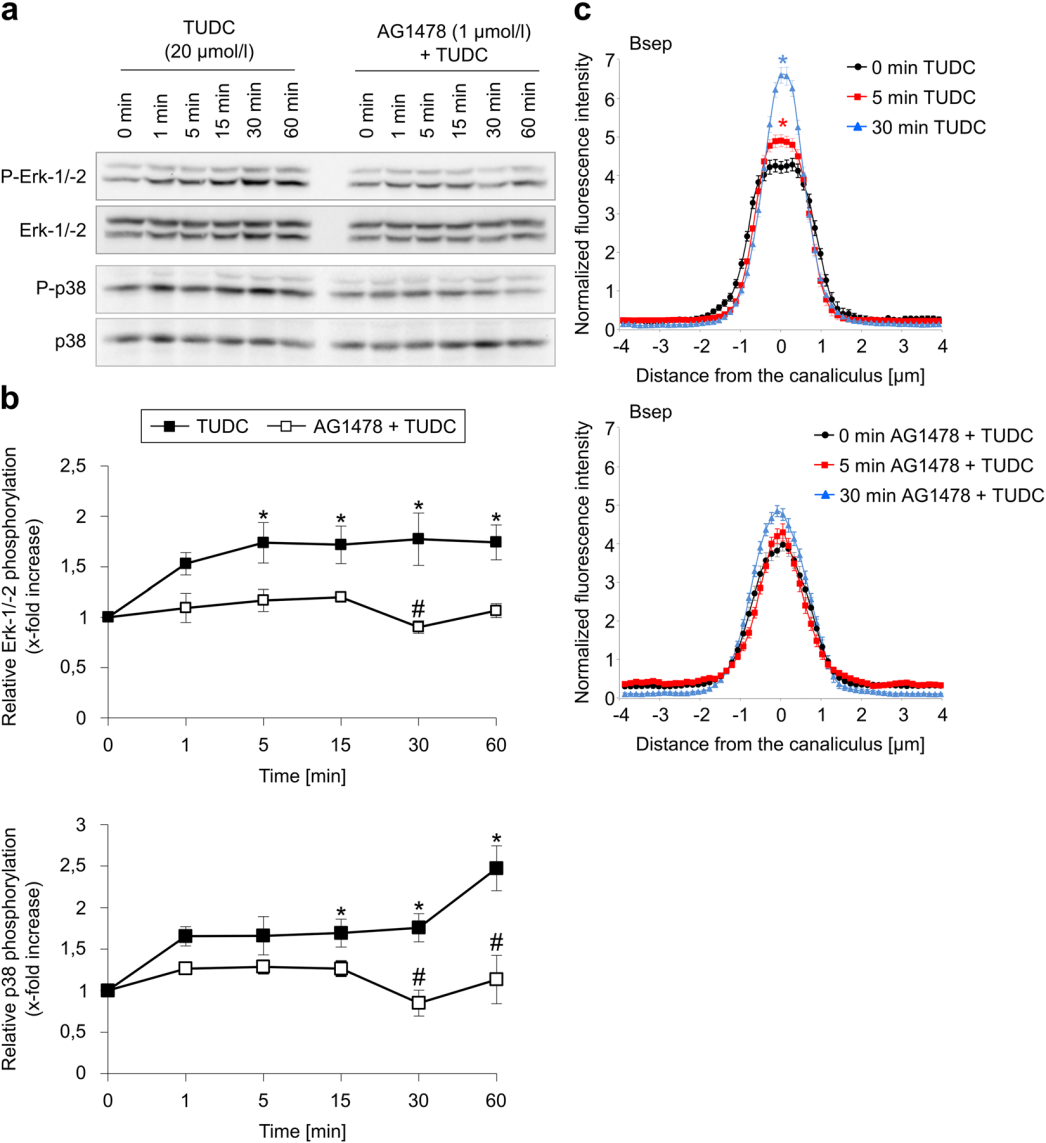
TUDC-induced dual activation of Erk-1/-2 and p38^MAPK^ and Bsep insertion into the canalicular membrane are dependent on EGFR phosphorylation. Rat livers were perfused with TUDC (20 µmol/l) for up to 60 min. When indicated, AG1478 (1 µmol/l) was added 30 min prior to TUDC to the perfusate. (**a**) Phosphorylation of Erk-1/-2 and p38^MAPK^ was analyzed by use of specific antibodies. Total Erk-1/-2 or p38^MAPK^, respectively, served as loading controls. (**b**) Western blots were analyzed densitometrically. Phosphorylation level at *t* = 0 min was set to 1. Representative blots and statistics (mean ± SEM) of at least three independent perfusion experiments are shown. TUDC induced a significant increase in Erk-1/-2 and p38^MAPK^ phosphorylation (*p < 0.05), which was significantly inhibited by AG1478 (#*p* < 0.05). (**c**) Cryosections from perfused rat liver were immunostained for Bsep and ZO-1 (see Supplementary Fig. 13), fluorescence images were recorded by confocal LSM (see Supplementary Fig. 13), and analyzed densitometrically. Blots were cropped to focus on the area of interest, and full-length blots are presented in Supplementary Figure 12. Under control conditions (black, *t* = 0 min), Bsep is largely localized between the linear ZO-1, but is also found inside the cells. Addition of TUDC (blue, *t* = 5 min; red, *t* = 30 min) results in *t*he insertion of intracellular Bsep into the canalicular membrane, which was inhibited by AG1478. The fluorescence profiles depicted are statistically significantly (*p* < 0.05) different from each other with respect to variance and peak height.

The choleretic action of TUDC is largely due to an Erk-1/-2- and p38^MAPK^-dependent insertion of the intracellularly stored canalicular transporters Bsep and Mrp2^10,13^ downstream of EGFR activation. The inhibitor of EGFR tyrosine kinase activity, AG1478, to a large extent abolished the TUDC-induced Erk-1/-2 and p38^MAPK^ activation (Fig. 8, Supplementary Fig. 12). Immunofluorescence stains of the canalicular bile salt transporter Bsep as well as the tight junction complex protein ZO-1, which delineates the bile canaliculi, were analyzed by CLSM and a densitometric analysis procedure^11,14–16^. In liver tissue, ZO-1 is arranged along two lines, and canalicular transporters within the canalicular membrane are located between these lines (see Supplementary Fig. 13). During control conditions, Bsep was located predominantly in the canalicular membrane (Supplementary Fig. 13). Densitometric analysis after perfusion with TUDC (20 µmol/l) revealed significantly different Bsep fluorescence profiles already after 5 min (Fig. 8)^10^ (*p* < 0.05; *F*-test for differences in peak heights and variances of Gaussian fits to the data sets) and a narrowing of the fluorescence signal by 0.4 ± 0.04 µm, i.e., by ~30%, (determined from the difference in the full width at half maximum (FWHM) values of the fitted Gaussians) after 30 min. Like Erk-1/-2 and p38^MAPK^ activation, Bsep insertion into the canalicular membrane was also inhibited by AG1478 (Fig. 8, FWHM_t=0 min_: 1.48 ± 0.03 µm vs. FWHM_t=30 min_: 1.51 ± 0.03 µm). ZO-1 immunostaining did not change under any condition (see Supplementary Fig. 13).

### norUDCA induces a transient insertion of Bsep into the canalicular membrane

**Figure 9:**
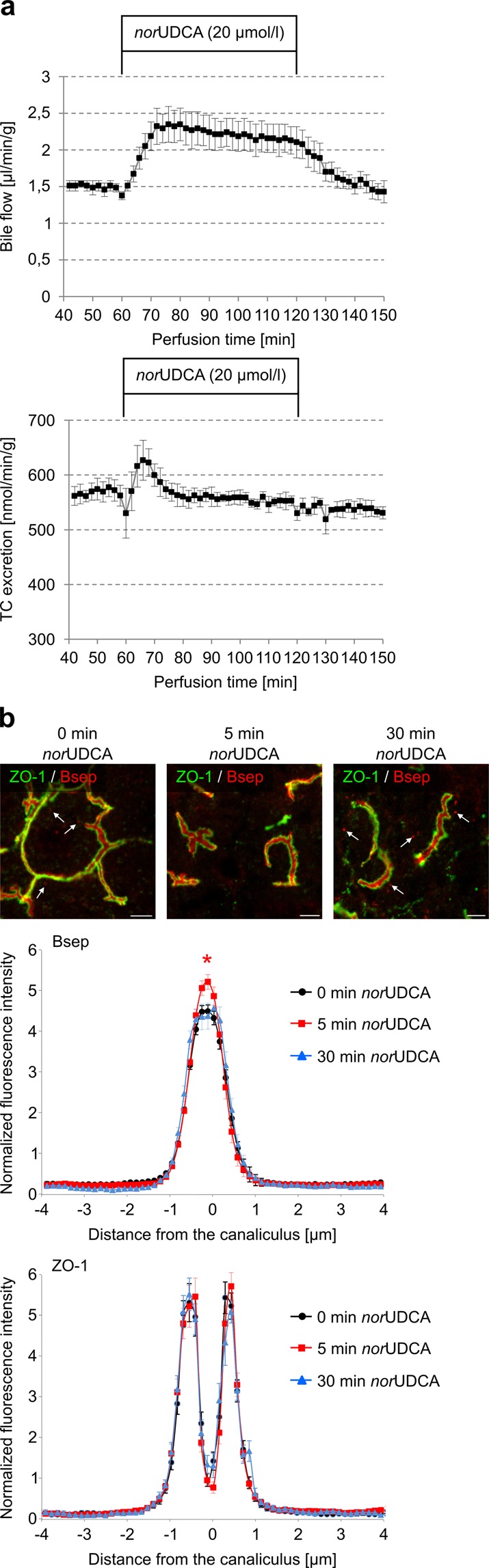
*nor*UDCA induced increased bile flow and TC excretion in perfused rat liver. (**a**) Livers were preperfused in the presence of 10 µmol/l [^3^H]TC. Data are given as mean ± SEM from four different experiments. After a pre-perfusion period of 20 min, *nor*UDCA (20 µmol/l) was added for 30 min. *nor*UDCA increased bile flow over the complete perfusion period and excretion of TC within the first 10 min of perfusion. (**b**) Cryosections from perfused rat liver were immunostained for Bsep and ZO-1, fluorescence images were recorded by confocal LSM, and analyzed densitometrically. Representative pictures of at least three independent experiments are depicted. The scale bar corresponds to 5 µm. Under control conditions (black, *t* = 0 min), Bsep is largely localized between the linear ZO-1, but is also found inside the cells (white arrows). *nor*UDCA (red, *t* = 5 min) led to the insertion of intracellular Bsep into the canalicular membrane. The fluorescence profiles depicted are statistically significantly (*p* < 0.05) different from each other with respect to variance and peak height. Under control conditions, ZO-1 fluorescence profiles show two peaks. Liver perfusion experiments with *nor*UDCA resulted in no significant changes of ZO-1 fluorescence profiles with respect to the distance of the peaks and the variance of fluorescence profiles. Means ± SEM of 30 measurements in each of at least three individual experiments for each condition are shown.

TUDC has been shown to increase the capacity for TC excretion into bile^10,17^. As shown in Fig. 9, *nor*UDCA (20 µmol/l) increased bile flow and stimulated a transient TC excretion within the first 10 min of perfusion, whereas the TUDC observed stimulation of TC excretion was prolonged^10^. Bsep is responsible for the bile salt-dependent bile flow and transports, among others, conjugates of cholic acid (CA) and chenodeoxycholic acid (CDCA), and the bile acid deoxycholic acid (DCA). In addition, it secretes ursodeoxycholic acid (UDCA) and its conjugates into bile^18^. Most of the Bsep immunofluorescence was found between the parallel rows of ZO-1 staining under control conditions, which indicates that Bsep is localized in the canalicular membrane. However, even under control conditions, there was some punctate Bsep staining in the cytosol, mainly in the subcanalicular region, suggestive for the presence of Bsep-containing vesicles inside the cell (Fig. 9). Addition of *nor*UDCA (20 µmol/l) results within 5 min in the disappearance of intracellular Bsep, and Bsep staining was almost exclusively found in the canaliculi (Fig. 9). This is reflected in the fluorescence profile, which shows a significant increase in canalicular Bsep fluorescence intensity after 5 min of *nor*UDCA addition (Fig. 9); in contrast to TUDC-induced Bsep insertion (Fig. 8), the increase vanished after 30 min, and a punctuated intracellular Bsep staining reappeared (Fig. 9). These findings suggest a *nor*UDCA-induced transient translocation and insertion of intracellular Bsep into the canalicular membrane. In contrast, *nor*UDCA has no effect on the distribution of the basolateral transporter Ntcp (see Supplementary Fig. 14). Subcellular Ntcp distribution in control and *nor*UDCA (20 µmol/l)-perfused livers was analyzed and quantified by CLSM and densitometric fluorescence intensity analysis as described in the Methods section. For labeling of the plasma membrane, liver sections were stained with a specific antibody against the plasma membrane marker protein Na^+^/K^+^-ATPase. The immunofluorescence analysis shows that there was no obvious change in Ntcp and Na^+^/K^+^-ATPase distribution at the basolateral membrane within 30 min (see Supplementary Fig. 14).

### TC inhibits norUDCA-induced α_5_β_1_ integrin activation

TC at a concentration of 100 µmol/l had no β_1_ integrin-activating activity but interfered with TUDC-induced α_5_β_1_ integrin activation^5^. Similarly, when *nor*UDCA was added on top of TC (100 µmol/l), active β_1_ integrin was barely detectable in isolated perfused rat liver (Supplementary Fig. 15). This indicates that TC interferes with *nor*UDCA-induced α_5_β_1_ integrin activation.

### TUDC and norUDCA bind directly to α_5_β_1_ integrin with similar affinities

Inhibition (i.e., IC_50_ values) of α_5_β_1_ integrin binding to immobilized fibronectin by TUDC and *nor*UDCA was determined using a standardized, competitive ELISA-based assay^19^ and Cilengitide^20^ as a control. TUDC and *nor*UDCA showed similar IC_50_ values in the low millimolar range (Table 1, Supplementary Fig. 16), demonstrating a similar binding affinity of both compounds and confirming that the observed activation of α_5_β_1_ integrin results from direct binding of the bile acids to the MIDAS site in the integrin head group.

**Table 1:** Affinities of TUDC and *nor*UDCA and the control peptide towards the RGD-recognizing integrin α_5_β_1_ obtained from an ELISA-like solid-phase binding assay.

Compound	Sequence	IC_50_^a^
**TUDC**	—	4.01 (1.80…8.98)^b^
***nor*UDCA**	—	3.93 (1.34…11.5)^b^
**Control peptide Cilengitide**	*c*(Arg-Gly-Asp-d-Phe-*N*Me-Val)	15.4 (14.49…16.36)^c^

^a^The IC50 values were obtained from a sigmoidal fit to two independent data rows (serial dilutions). The 95% confidence interval is given in brackets.

^b^In mM.

^c^In nM.

## Discussion

In this study, we addressed the question to what extent side chain-modified derivatives of TUDC (*nor*UDCA, T*nor*UDCA, GUDC, UDCA) can directly activate α_5_β_1_ integrin and whether the signaling events downstream of integrin activation differ from those triggered by TUDC.

Applying all-atom MD simulations, the potential activity of *nor*UDCA, T*nor*UDCA, GUDC, and UDCA was assessed on the basis of three geometric parameters, and compared to that of TUDC and TC investigated previously^5,7^. The geometric parameters were derived from crystal structures of the closed (PDB: 3FCU) and open (PDB: 3FCS) α_IIb_β_3_ integrin headpiece (Fig. 2b)^21^, as well as based on previous simulation results^5^: the α1 kink angle, the α7 tilt angle and the propeller-βA distance. Although the crystal structure of the open α_5_β_1_ headpiece has remained elusive, it is likely that the conformational changes involved in α_5_β_1_ integrin activation are very similar to those observed for other integrin subtypes (Supplementary Fig. 17)^22,23^. Among the six bile acids tested, TUDC- and *nor*UDCA-bound structures displayed on average significantly higher values for all three geometric parameters (Fig. 2, Supplementary Table 3). T*nor*UDCA- and GUDC-bound integrin displayed a larger α1 kink angle and, especially, α7 tilt angle than integrin bound to UDCA and TC but the propeller-βA distance was similar among all four of these bile acids. Hence, we classified TUDC and *nor*UDCA as highly activating, T*nor*UDCA and GUDC as weakly activing, and UDCA and TC as inactive or inhibitory ligands, respectively. Note that larger conformational changes in the α_5_β_1_ integrin ectodomain, which have been linked to integrin activation^21,23–25^, cannot be expected to be observed during our sub-μs long MD simulations compared to integrin activation times *in vivo*^26,27^.

To evaluate the robustness of the predictions from our MD simulations, we correlated the mean values of the three geometric parameters measured in each triplet of MD simulations against the rank of the bile acids in terms of their activity (Fig. 2), as deduced from the amount of immunostained, active β_1_ integrin induced by the respective bile acid (Fig. 3). Accordingly, TUDC is the most active bile acid, followed by *nor*UDCA, T*nor*UDCA, GUDC, UDCA, and TC. We obtained significant correlations between the average α1 kink angle (R^2^ = 0.66, *p* = 0.05), or the α7 tilt angle (R^2^ = 0.83, *p* = 0.01), and the rank (Fig. 2). Thus, the set of geometric parameters used for the analysis of the MD simulations was not only capable to distinguish between active and inactive bile acids but also captured more subtle differences in the activities. Therefore, in future studies, such MD simulations might serve as a “computational assay” to test potential candidate molecules for their ability to activate α_5_β_1_ integrin.

As predicted by MD simulations, *nor*UDCA caused a dose-dependent activation of α_5_β_1_ integrins in hepatocytes (Fig. 3a), and this dose-dependent activation is weaker than the one observed with TUDC (Fig. 4a)^5^: While after addition of TUDC the active conformation of the β_1_ integrin subunit becomes markedly visible within 1 min, *nor*UDCA reaches a similar extent of β_1_ integrin activation after 15 min (Figs. 3a and 4a). A standardized, competitive ELISA-based solid-phase assay revealed that TUDC and *nor*UDCA directly bind to the MIDAS site in the integrin head group, confirming that the observed activation of α_5_β_1_ integrin results from direct binding of the bile acids, and that the binding affinities of both compounds are similar (Table 1, Supplementary Fig. 16). The latter finding, together with using for TUDC and *nor*UDCA the same concentrations in all experiments, rules out that the different extent of activation of α_5_β_1_ integrin by the bile acids is caused by differential occupation of the binding site. The low affinities of both compounds are concordant with the fact that the compounds do not activate α_5_β_1_ integrin when located in the plasma membrane; extracellular TUDC and *nor*UDCA concentrations in the perfusion experiments were at most 50 μM. Ntcp-transfected HepG2 cells stimulated with a TUDC concentration of 100 μM do not show active β_1_ integrin in the cell membrane either^5^. In contrast, intracellular bile acid concentrations can reach single digit mM concentrations, as estimated from intracellular bile acid contents for hepatocyte cultures^28^ or rat hepatoma cells^29^. The uncertainty in estimating intracellular bile acid concentrations is reflected, however, in that measurements of bile acid concentrations in human liver tissue^30^ together with those of intracellular water space in rat liver^31^ yielded bile acid concentrations about one order of magnitude smaller than the IC_50_ values. Finally, with respect to whether the low affinities might be indicative of non-specific binding, note that both RGD peptides and TC inhibit TUDC-induced activation of α_5_β_1_ integrin and the signal transduction pathways following integrin activation^5^. Here, we show that this also applies to *nor*UDCA-induced activation of α_5_β_1_. We consider particularly the inhibitory effect of TC with respect to TUDC a consequence of competitive antagonism at the MIDAS because we find it difficult to grasp how two bile acids with very similar structures could cause opposing effects via nonspecific mechanisms.

Although the results of our MD simulations indicate that *nor*UDCA is less potent than TUDC with respect to direct α_5_β_1_ integrin activation, additional kinetic reasons may contribute as well to this difference. *nor*UDCA, unlike TUDC, is not readily taken up into the hepatocyte via Ntcp or other transport systems^32^, and the transbilayer transport rate of *nor*CDCA, an epimer of *nor*UDCA, is six-fold higher than of CDCA^33^, suggesting that *nor*UDCA is passively transported across the sinusoidal membrane. Slow, passive sinusoidal uptake would then be opposed by a fast, active outward transport by a canalicular transporter, presumably Mrp2^34–37^. Depending on the rates, this situation might prevent concentrating *nor*UDCA inside the hepatocyte. For TUDC, a concentrative uptake into the hepatocyte was proposed as a likely requirement for α_5_β_1_ integrin activation^5^.

TUDC-mediated integrin activation is followed by a *sustained* dual activation of Erks and p38^MAPK^, which is the crucial downstream signaling event towards choleresis^10,17^. Such a sustained activation of Erks also occurs with lower and higher concentrations (10 µmol/l and 50 µmol/l) of TUDC (Supplementary Fig. 18), rendering a concentration effect unlikely. *nor*UDCA also induced a similar but only *transient* dual activation of these MAPKs, which was sensitive to integrin inhibition by an RGD motif-containing hexapeptide (Figs. 5a,b and 6b, Supplementary Figs. 5, 6, 10). This transient MAPK activation might be a consequence of the weaker activation of α_5_β_1_ shown above. As *nor*UDCA-induced Erk-1/-2 phosphorylation was not amplified when phosphatases were inhibited with okadaic acid (Supplementary Fig. 4), it is unlikely that the transient MAPK activation by *nor*UDCA is mediated via activation of phosphatases. In this context, note that perfusion with TUDC caused a significant EGFR/c-Src association after 15 min (Fig. 7, Supplementary Fig. 11). By contrast, such an association was not observed following perfusion with *nor*UDCA (Fig. 7, Supplementary Fig. 11). Taken together, our results thus suggest that a c-Src-dependent trans-activation of the EGFR is central for a sustained MAPK activation. At first glance, the suggested sustainer role of EGFR appears contradicted by the observation that AG1478, a selective inhibitor of EGFR tyrosine kinase activity^38^, abolished the TUDC-induced phosphorylation of Erk and p38^MAPK^ (Fig. 8, Supplementary Fig. 12): If EGFR activation only *sustained* Erk activation, EGFR inhibition should not decrease the extent of TUDC-mediated Erk activation, but only change its time course. However, AG1478 treatment has been shown to compromise basal levels of EGFR phosphorylation^39^, and such a basal EGFR activity was suggested to be required for proper MAPK signaling^40^.

In line with the transient or sustained character of *nor*UDCA-mediated and TUDC-mediated MAPK activation, respectively, *nor*UDCA induced only a transient insertion of intracellular Bsep into the canalicular membrane, whereas TUDC-induced insertion of Bsep was sustained (Figs. 8 and 9). In line with this, *nor*UDCA only transiently increased TC excretion into bile (Fig. 9a). However, as expected, *nor*UDCA increased bile flow in a sustained way due to *nor*UDCA excretion into bile and induction of a bicarbonate-rich hypercholeresis^41,42^. Dual activation of Erks and p38^MAPK^ is required for the TUDC-induced stimulation of Bsep insertion into the canalicular membrane^10^. In line with this, inhibition of EGFR tyrosine kinase activity by AG1478 prevented Bsep insertion during perfusion with TUDC (Fig. 8c), again suggesting that (at least basal) EGFR activity is an essential requirement for dual MAPK signaling towards choleresis.

Inhibition of PI3-K by wortmannin abolished the *nor*UDCA-induced phosphorylation of Erks, but not of p38^MAPK^, while inhibition of c-Src by PP-2 abolished phosphorylation of Erks and p38^MAPK^, suggesting that c-Src activation lies upstream of PI3-K activation. The PI3-K/Ras/Erk pathway has been described as essential for the choleretic effect of TUDC^43^. Whether c-Src directly activates PI3-K^44^, or indirectly via EGFR, was not addressed in this study. An earlier study suggested that genistein-sensitive tyrosine kinases such as EGFR are not involved in the activation of the PI3-K/Ras/Erk pathway by TUDC^43^. However, whether the Ras/Erk pathway becomes PI3-K-dependent also depends on the extent of EGFR activation^45^.

**Figure 10:**
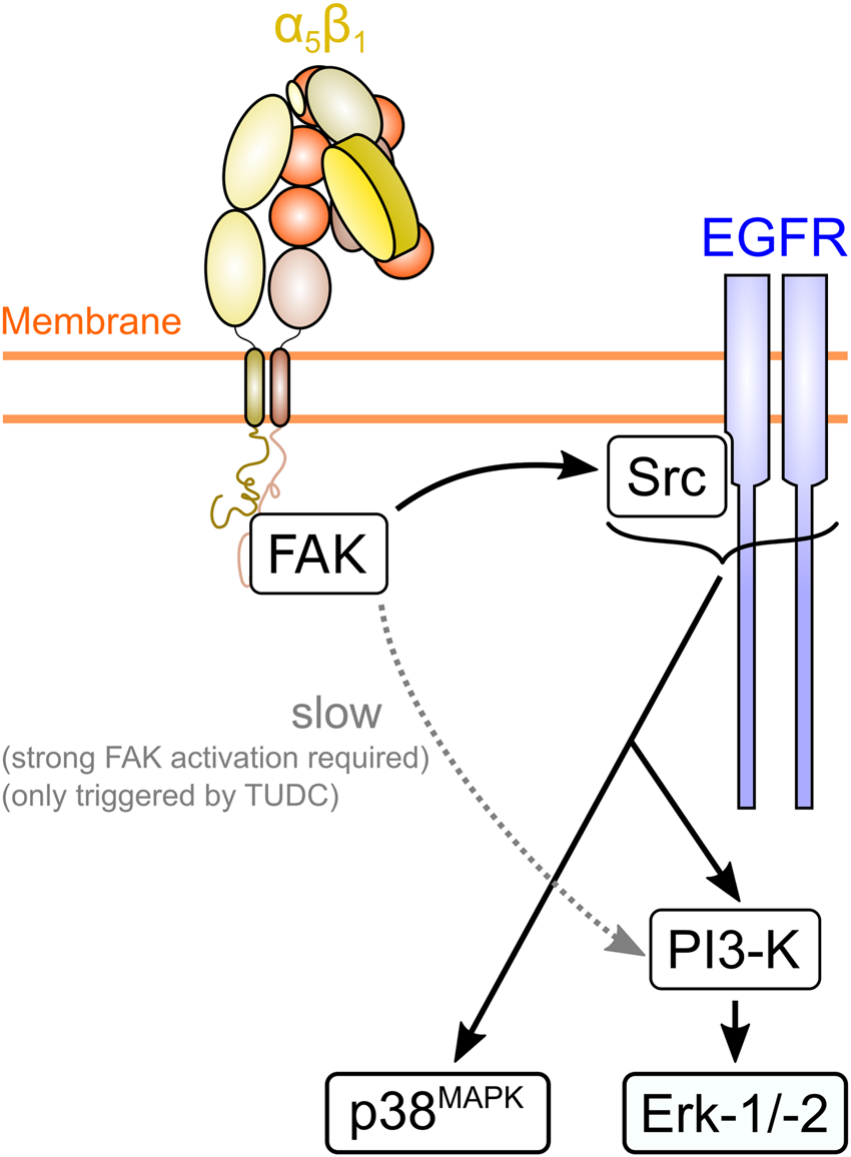
Model of α_5_β_1_ integrin activation-dependent differential bile acid signaling. Activation of α_5_β_1_ integrin with the less efficacious *nor*UDCA results in the formation of FAK^Y397-P^, which leads to c-Src- and PI3-K-dependent Erk-1/2 activation. When α_5_β_1_ integrin is activated by the more efficacious TUDC, higher levels of FAK^Y397-P^ result, which, in addition, trigger a slower activation of Erk-1/-2 via PI3-K in a c-Src-independent manner^7^.

Notably, in an earlier study with TUDC^7^, inhibition of c-Src did not prevent Erk-1/-2 activation, but only delayed it by ~8 min. Hence, in view of the above results, inhibition of c-Src activity by PP-2 seems to prevent Erk-1/-2 activation only when *nor*UDCA is used as an integrin agonist. Based on our and literature data, we therefore suggest the following ligand-dependent selectivity for signaling pathways induced by α_5_β_1_ integrin (Fig. 10): One of the first steps in integrin-mediated signaling is the recruitment of focal adhesion kinase (FAK)^46^ and its subsequent autophosphorylation, an event also observed during TUDC-mediated activation of α_5_β_1_ integrin^7^. Levels of autophosphorylated FAK (FAK^Y397-P^) were shown to increase linearly with the amount of fibronectin-bound (i.e. active, signaling-competent) α_5_β_1_^47^. Thus, a highly efficacious integrin activation as observed with TUDC^5^ would result in high FAK^Y397-P^ levels, whereas a less efficacious integrin activation as observed with *nor*UDCA (this study) would result in lower FAK^Y397-P^ levels, as confirmed by densitometric analysis (Supplementary Fig. 8). FAK^Y397-P^ activates c-Src^48,49^, which in turn phosphorylates EGFR, and both the activated c-Src and EGFR mediate PI3-K activation^48,50^ and subsequent phosphorylation of Erk-1/-2. However, FAK^Y397-P^ can also *directly* activate PI3-K, independent of c-Src and the EGFR^48^. We now speculate that this direct, FAK-mediated activation of PI3-K is slower than the c-Src and EGFR-mediated PI3-K activation, and that only high FAK^Y397-P^ levels trigger this slow pathway. Hence, even when c-Src activity is inhibited by PP-2, a highly efficacious integrin activation by TUDC would lead to a pronounced FAK autophosphorylation and rescue Erk-1/-2 phosphorylation via a direct PI3-K activation, albeit with a time delay, as observed previously^7^. In contrast, a less efficacious integrin activation by *nor*UDCA would lead to less FAK autophosphorylation and, thus, require activated c-Src in order to switch on the then necessary PI3-K signal to activate Erk-1/-2, which would occur more rapidly (this study). According to this model, inhibition of EGFR activity by AG1478 should not abolish the Erk response, if TUDC-mediated PI3-K activation occurred via the slow pathway. Regarding the above observation that AG1478 did abolish the TUDC-induced phosphorylation of Erk and p38^MAPK^, we can only speculate at present that apparently (at least a basal) EGFR activity is required for PI3-K to properly function in this pathway, although the details of this interplay remain elusive.

Taken together, we demonstrated - to our knowledge for the first time - that *nor*UDCA directly activates α_5_β_1_ integrins in hepatocytes and triggers short-term choleresis via a transient activation of MAPKs followed by a transient insertion of Bsep into the canalicular membrane in addition to the known bicarbonate-rich hypercholeresis. Furthermore, we provide evidence that TUDC and *nor*UDCA exert a functional selectivity for certain signal transduction pathways in α_5_β_1_ integrin, a property – to our knowledge – not yet described for ligands interacting with integrins lacking an αI domain^3^. This functional selectivity may also provide a rationale for the differential therapeutic use of UDCA (which *in vivo* is rapidly conjugated to TUDC) and *nor*UDCA (which is resistant to amidation with taurine) in primary biliary cholangitis (PBC) and primary sclerosing cholangitis (PSC), respectively^42^. Although both compounds trigger hypercholeresis, the underlying mechanisms are different. TUDC induces choleresis by stimulating hepatocellular bile acid secretion, whereas *nor*UDCA induces a bicarbonate-rich hypercholeresis by cholehepatic shunting, but has no effect on hepatocellular bile acid secretion^41,51^. This may explain why *nor*UDCA is superior to UDCA in the treatment of sclerosing cholangitis in Mdr2 knockout mice^52^.

## Materials and Methods

### Materials

The materials used were purchased as follows: Ocadaic acid was from Enzo Life Sciences (Lörrach, Germany). PP-2, H-Gly-Arg-Gly-Asp-Ser-Pro-OH (G*RGD*SP), and H-Gly-Arg-Ala-Asp-Ser-Pro-OH (G*RAD*SP) were from Merck-Millipore (Darmstadt, Germany), FITC-coupled phalloidin, collagenase, insulin, and TUDC from Sigma Aldrich (Munich, Germany), penicillin/streptomycin and Fluoromount-G from Tocris/Biozol (Eching, Germany), fetal bovine serum (FBS) from Life Technologies GmbH (Darmstadt, Germany), cOmplete^TM^ protease inhibitor cocktail tablets and PhosSTOP^TM^ phosphatase inhibitor cocktail tablets were from Roche Diagnostics (Mannheim, Germany) and William’s Medium E from Biochrom (Berlin, Germany). *nor*UDCA was kindly provided by Dr. Falk Pharma (Freiburg, Germany). The Bsep antibody (K24)^53^ and the Ntcp antibody (K4)^54^ were generous gifts from Prof. Dr. B. Stieger (Kantonsspital Zürich, Switzerland). Antibodies recognizing zona occludens-1 (ZO-1, #33–9100), phospho-EGFR Tyr^845^ (#44–784), phospho-EGFR Tyr^1173^ (#44–794 G), and c-Src (#44–656) were from Life Technologies GmbH (Darmstadt, Germany). The antibodies raised against the α_5_β_1_ integrin dimer (AB1950) and the β_1_ integrin subunit active conformation (#MAB2079Z), phospho-Erk-1/-2 (#9106), phospho-p38^MAPK^ (#9211), p38^MAPK^ (#9228), phospho-EGFR Tyr^1045^ (#2237), phospho-Src-Tyr^418^ (#2101), phospho-FAK Tyr^925^ (#3284), and phospho-FAK Tyr^576/577^ (#3281) were from Cell Signaling Technology, Inc. (Danvers, USA), against Erk-1/-2 (#06–182), EGFR (#06–847, Western blot, WB), Na^+^/K^+^-ATPase (#05–369), Cy3-conjugated donkey anti-rabbit IgG (#AP182C), and FITC-conjugated donkey anti-mouse IgG (#AP192C) from Merck-Millipore (Darmstadt, Germany). The antibody against EGFR (sc-03) for immunoprecipitation (IP) studies was from Santa Cruz Biotechnology (Heidelberg, Germany). The polyclonal antibodies against phospho-FAK Tyr^397^ (#44–650 G)_,_ phospho-FAK Tyr^407^ (#44–624 G) and phospho-FAK Tyr^861^ (#44–626 G) were purchased from Thermo Fischer Scientific (Waltham, Massachusetts, USA). The monoclonal antibody against glyceraldehyde-3-phosphate dehydrogenase (GAPDH) was from Biodesign International (Saco, Maine, USA). Horseradish peroxidase-conjugated anti-mouse IgG (#1706516) and anti-rabbit IgG (#1721019) were from Bio-Rad Laboratories (Munich, Germany) and Dako (Hamburg, Germany). All other chemicals were from Merck-Millipore (Darmstadt, Germany) at the highest quality available.

### Generation of α_5_β_1_ integrin-bile acid complex structures and molecular dynamics simulations

A detailed description of how the starting structures for the MD simulations of α_5_β_1_ integrin bound to either TUDC, *nor*UDCA, T*nor*UDCA, GUDC, UDCA, or TC were generated and how MD simulations of in total 3.6 µs length of these systems were performed is provided in the Supplementary Text.

### Analysis of trajectories from molecular dynamics simulations

MD trajectories were visually inspected for conformational changes in VMD^55^. Conformational changes that may result in integrin activation were evaluated based on three geometric parameters (Fig. 1a,b): Straightening of the α1 helix, tilting of the α7 helix, and the distance between the β-propeller domain in the α-subunit and the βA domain in the β-subunit. Straightening of the α1 helix was monitored through an increase of its kink angle (Fig. 1b). During α_IIb_β_3_ integrin activation, this angle increases from ~144° to ~166°, as observed in crystal structures of the closed (PDB: 3FCU) and open (PDB: 3FCS) integrin^21^. Tilting of α7 was measured as the angle between the three points 1) ion at the “Adjacent to MIDAS” (ADMIDAS) site, 2) center of mass of the C_α_ atoms of the first four residues of the α7 helix, and 3) center of mass of the C_α_ atoms of the last four residues of the α7 helix (Fig. 1B). Upon activation of α_IIb_β_3_ integrins, the α7 helix pivots laterally^56^ (increase of the α7 tilt angle from ~128° to ~133°), accompanied by a marked increase of B-factors in the region of the α7 helix^23^ (Supplementary Fig. 17). A larger tilt angle of the α7 helix thus represents a defined, activating conformational change, as does the observation of a higher helix mobility, which is required for subsequent steps in integrin activation. Finally, the distance of the centers of mass of the propeller domain in the α subunit and the βA domain in the β subunit was measured, as it had been shown to increase during TUDC-induced α_5_β_1_ integrin activation^5^. All MD trajectory analyses were performed using the programs ptraj from AmberTools 1.5 or cpptraj from AmberTools13^57^.

### Liver perfusion

Livers from male Wistar rats (140–160 g) were perfused in a non-recirculating manner as described previously^58^. As a perfusion medium, the bicarbonate-buffered Krebs-Henseleit saline plus l-lactate (2.1 mmol/l) and pyruvate (0.3 mmol/l) gassed with 5% CO_2_ and 95% O_2_ at 37 °C was used (305 mosmol/l, normoosmotic). Inhibitors and bile acids were added to the influent perfusate by dissolution into the Krebs-Henseleit buffer. Viability of the perfused livers was assessed by measuring lactate dehydrogenase leakage into the perfusate. The portal pressure, the effluent K^+^ concentration, and pH were continuously monitored. In bile formation experiments, livers were perfused with 10 μmol/l [^3^H] taurocholate (1 μCi/l). Bile was collected at intervals of 2 min. Bile flow was assessed by gravimetry, assuming a specific mass of 1 g/ml. Taurocholate excretion into bile was determined by liquid scintillation counting of the radioactivity present in bile, based on the specific radioactivity of [^3^H] taurocholate in influent perfusate. To wash out endogenously formed bile acids and obtain a steady-state TC excretion, livers were preperfused for 20 min before experimental maneuvers were started. All experiments were approved by the responsible local authorities of the “Zentrale Einrichtung für Tierforschung und wissenschaftliche Tierschutzaufgaben” (ZETT) of the University of Düsseldorf and the “Landesamt für Natur, Umwelt und Verbraucherschutz Nordrhein-Westfalen” (LANUV, NRW) (file number: 84.02-04.2012A214). We confirm that all experiments were performed in accordance with relevant guidelines and regulations.

### Immunofluorescence staining

Immunofluorescence staining was performed as described before^5,16^ (see Supplementary Text for a detailed protocol).

### Densitometric fluorescence intensity analysis

(see Supplementary Text for a detailed protocol).

### Immunoblot analysis

Immunoblot analysis was performed as described before^16^ (see Supplementary Text for a detailed protocol).

### Immunoprecipitation

Immunoprecipitation was performed as described before^16^ (see Supplementary Text for a detailed protocol).

### Integrin binding assay

The affinity and selectivity of bile acid derivatives were determined by a solid-phase binding assay applying a previously described protocol^19^ that involves coated extracellular matrix proteins and soluble integrins. Cilengitide^20^ (*c*(f(*N*Me)VRGD) (α_5_β_1_: IC_50_ = 15.4 nM) was used as internal standard. Flat-bottomed 96-well ELISA plates (BRAND, Wertheim, Germany) were coated overnight at 4 °C with ECM protein (1) (100 *μ*L per well) in carbonate buffer (15 mM Na_2_CO_3_, 35 mM NaHCO_3_, pH 9.6). Each well was then washed with PBS-T buffer (phosphate-buffered saline/Tween 20, 137 mM NaCl, 2.7 mM KCl, 10 mM Na_2_HPO_4_, 2 mM KH_2_PO_4_, 0.01% Tween 20, pH 7.4; 3 × 200 *µ*L) and blocked for 1 h at room temperature (RT) with TS-B buffer (Tris-saline/bovine serum albumin (BSA) buffer, 20 mM Tris-HCl, 150 mM NaCl, 1 mM CaCl_2_, 1 mM MgCl_2_, 1 mM MnCl_2_, pH 7.5, 1% BSA; 150 *μ*L/well). Meanwhile, a dilution series of the compound and internal standard was prepared in an extra plate, ranging from 66 mM to 58 *µ*M. After washing the assay plate three times with PBS-T (200 *μ*L), 50 *μ*L aliquots of the dilution series were transferred to each well from B-G in six appropriate concentrations. Well A was filled with 100 *μ*L of TS-B buffer (blank), and well H was filled with 50 *μ*L of TS-B buffer. Then, 50 *μ*L of a solution of human integrin (2) in TS-B buffer was transferred to wells H–B and incubated for 1 h at RT. The plate was washed three times with PBS-T buffer, and then primary antibody (3) (100 *μ*L per well) was added to the plate. After incubation for 1 h at RT, the plate was washed three times with PBS-T. Then, secondary peroxidase-conjugated antibody (**4**) (100 *µ*L/well) was added to the plate and incubated for 45 min at RT. The plate was then washed three times with PBS-T, developed by the addition of SeramunBlau (50 *μ*L/well, Seramun Diagnostic GmbH, Heidesee, Germany) and incubated for approx. 1 min at RT in the dark. The reaction was stopped with 3 M H_2_SO_4_ (50 *µ*L/well), and the absorbance was measured at 450 nm with a plate reader (infinite M200 Pro, TECAN). The IC_50_ value (with 95% confidence interval) of each compound resulted from a sigmoidal fit to 32 data points, obtained from two serial dilution rows, by using the GraphPad Prism software package. All IC_50_ values determined were referenced to the affinity of the internal standard.

1. 0.5 μg mL^−1^, human fibronectin, Sigma-Aldrich.
2. 2.0 μg mL^−1^, human α_5_β_1_-integrin, R&D.
3. 1.0 μg mL^−1^, mouse anti-human CD49e, BD Biosciences.
4. 2.0 μg mL^−1^, anti-mouse IgG-POD, Sigma-Aldrich.

### Statistical analysis

Statistical analysis of the data from MD simulations was performed in R^59^. Mean values and their respective standard errors were computed using the last 100 ns of each simulation. The statistical significance of differences in simulation means was assessed by Student’s t-test. *p* < 0.05 was considered statistically significant.

As to experimental work, unless stated otherwise in the respective subsections of the Materials and Methods section, results from at least three independent experiments are expressed as mean values ± SEM. *n* refers to the number of independent experiments. Differences between experimental groups were analyzed by Student’s t-test, one-way analysis of variance following Dunnett’s multiple comparison post hoc test, or two-way analysis of variance following Bonferroni’s multiple comparison post hoc test where appropriate (GraphPad Prism; GraphPad, La Jolla, USA; Microsoft Excel for Windows). *p* < 0.05 was considered statistically significant.

## Supplementary information

Supplementary Information.

## Data Availability

All data generated or analyzed during this study are included in this published article (and its Supplementary Information file).
