# Supplementary material for: Evidence for functional selectivity in TUDC- and norUDCA-induced signal transduction via α5β1 integrin towards choleresis
Source: Sci Rep. 2020 Apr 2;10:5795. doi: 10.1038/s41598-020-62326-y (PMC7118123; doi:10.1038/s41598-020-62326-y)
Supplement: Supplementary file 1 — Supplementary Information. [file 41598_2020_62326_MOESM1_ESM.pdf]

Supplementary Information

Evidence for functional selectivity in TUDC- and *nor*UDCA-induced signal transduction via $\alpha_5\beta_1$ integrin towards choleresis

Michele Bonus[1], Annika Sommerfeld[2], Natalia Qvartskhava[2], Boris Görg[2], Beatrice Stefanie Ludwig[3], Horst Kessler[3], Holger Gohlke[1,4], Dieter Häussinger[2]**

[1]Institute for Pharmaceutical and Medicinal Chemistry, Department of Mathematics and Natural Sciences, Heinrich Heine University Düsseldorf, Universitätsstr. 1, 40225 Düsseldorf, Germany

[2]Clinic for Gastroenterology, Hepatology and Infectious Diseases, Heinrich Heine University Düsseldorf, Moorenstr. 5, 40225 Düsseldorf, Germany

[3]Institute for Advanced Study and Center for Integrated Protein Science, Department of Chemistry, Technische Universität München, Lichtenbergstr. 4, 85747 Garching, Germany

[4]John von Neumann Institute for Computing (NIC), Jülich Supercomputing Centre (JSC), and Institute for Complex Systems - Structural Biochemistry (ICS-6), Forschungszentrum Jülich GmbH, Wilhelm-Johnen-Straße, 52425 Jülich, Germany

Table of Contents

Suppl. Text 3

Suppl. Figure 1: Primary and conjugated bile acids 7

Suppl. Figure 2: Structural stability during the MD simulations 8

Suppl. Figure 3: Effects of *nor*UDCA, T*nor*UDCA, GUDC and UDCA on β_1 integrin activation 9

Suppl. Figure 4: Effects of OA on *nor*UDCA- and TUDC-induced Erk-1/-2 activation 10

Suppl. Figure 5: *nor*UDCA-induced activation of Erk-1/-2, $p38^{MAPK}$ and Src 11

Suppl. Figure 6: *nor*UDCA-induced activation of Erk-1/-2, $p38^{MAPK}$ and Src 12

Suppl. Figure 7: Control perfusion experiments 13

Suppl. Figure 8: Phosphorylation of FAK^{Y397} in *nor*UDCA- and TUDC-perfused rat livers 14

Suppl. Figure 9: Phosphorylation of $FAK^{Y925,861,576/577,407}$ in perfused rat livers 15

Suppl. Figure 10: *nor*UDCA- and TUDC-induced Erk-1/-2, $p38^{MAPK}$ and EGFR activation 17

Suppl. Figure 11: *nor*UDCA- and TUDC-induced c□Src activ. and EGFR/c□Src association 18

Suppl. Figure 12: TUDC-induced dual activation of Erk-1/-2 and $p38^{MAPK}$ 19

Suppl. Figure 13: Immunohistochemical determination of Bsep and ZO-1 distribution 20

Suppl. Figure 14: Regulation of Ntcp in *nor*UDCA-perfused rat liver 21

Suppl. Figure 15: Inhibition of *nor*UDCA-induced β_1 integrin activation by TC 22

Suppl. Figure 16: Binding of TUDC and *nor*UDCA to $\alpha_5\beta_1$ integrin 23

Suppl. Figure 17: Flexibility changes in the βA domain upon activation 24

Suppl. Figure 18. Comparison between *nor*UDCA- and TUDC-induced Erk-1/-2 activation 25

Suppl. Figure 19. Subunit and domain organization of the $\alpha_5\beta_1$ integrin ectodomain 26

Suppl. Table 1: Domain-wise minimum, maximum and average RMSD values 27

Suppl. Table 2: Angles and distances computed from MD trajectories 28

Suppl. Table 3: Statistical testing for differences in mean values 29

Suppl. References 30

Note: some figure titles in the table of contents were shortened for reasons of clarity.

Supplementary Text

Materials and Methods

Generation of $\alpha_5\beta_1$ integrin-bile acid complex structures

To generate starting structures for the MD simulations, a 3D model of the $\alpha_5\beta_1$ integrin ectodomain was used as the receptor. This model had been generated by us by homology modeling in a previous study[1]. The same model had been used in the study identifying TUDC as a potent activator of $\alpha_5\beta_1$ integrin-mediated signaling[2].

3D structures of the bile acids *nor*UDCA, T*nor*UDCA, GUDC, and UDCA were generated with Maestro[3] and prepared with the LigPrep suite of programs[4]: I) Protonation states for each bile acid were calculated at pH 7.4 ± 0.2 using Epik[5]. II) Energy-minimized ring conformations were obtained via ring sampling from the template collection embedded in the ring_conf utility. III) Each bile acid was energy-minimized using the premin tool in LigPrep and bmin in MacroModel[6].

To generate bound conformations of each bile acid in the binding site of $\alpha_5\beta_1$ integrin, the conformation of TUDC in the ligand-bound homology model was used as a template for structural alignment of the other bile acids with the program Moloc. To maintain coordination to the Mg^{2+} ion in the binding site, the acidic moieties and the side chains of each bile acid were first superimposed on the sulfonate group and the side chain of TUDC. Second, the 5β-androstane core of T*nor*UDCA and GUDC was superimposed onto the 5β-androstane core of TUDC. This procedure was omitted for *nor*UDCA and UDCA, because the side chains in these bile acids are not sufficiently long to allow superposition of the core. Instead, the orientation of the 5β-androstane core of these bile acid was manually adjusted to resemble its orientation in TUDC. Finally, the ligand poses were energy-minimized in the receptor with the MAB force field[7].

MD simulations

All MD simulations were set up and performed with the AMBER 11 suite of programs[8] using the ff99SB force field[9] for the protein and the general AMBER force field (GAFF)[10] for the bile acids. Parameters from Åquist *et al.*[11] were used to treat the Mg^{2+} ions. Partial charges for the ligand atoms were derived according to the RESP procedure[12]. Each integrin-bile acid complex

was neutralized by addition of Na^+ ions and solvated in an octahedral periodic box of TIP3P water molecules[13]. The closest allowed distance between the outermost atom of the protein and the border of the periodic box was 11 Å. The Particle Mesh Ewald method[14] was used to calculate long-range electrostatic interactions. For each simulation, a 2 fs integration time step was used, and the cutoff distance for direct space non-bonded interactions was set to 8 Å.

For thermalizing the systems, solute atoms were restrained by harmonic positional restraints with a force constant of 25 $kcal \cdot mol^{-1} \cdot Å^{-2}$, and the systems were energy-minimized for 50 steps with the conjugate gradient method, followed by 200 steps with the steepest descent method. This procedure was repeated with a lower force constant of 5 $kcal \cdot mol^{-1} \cdot Å^{-2}$. While keeping the restraints on the solute atoms, gradual heating from 100 K to 300 K was performed for 40 ps in the canonical (NVT) ensemble. Target temperature and restraints were kept for another 10 ps. To adjust the solvent density, the systems were subjected to three consecutive restrained simulations of 50 ps length in the isothermal-isobaric (NPT) ensemble. Gradual removal of the restraints was performed during 100 ps of NVT-MD. The systems were then subjected to 200 ns of NVT-MD, with coordinates extracted every 20 ps. We performed three independent simulations of 200 ns length of the $\alpha_5\beta_1$-bile acid complexes containing *nor*UDCA, T*nor*UDCA, GUDC, and UDCA, and two additional independent simulations of $\alpha_5\beta_1$ integrin bound to TUDC and TC. In addition, for TUDC and TC, data of MD simulations of 200 ns length was reused from ref.[2].

Immunofluorescence staining

Cryosections of liver samples (7 µm) were either fixed in methanol (2 min, -20°C) or fixed and permeabilized with Zamboni (20 min, room temperature) and Triton X-100 (2 min, 4°C). Then, liver sections were blocked with FBS (5% (*w/v*), 30 min, room temperature) and incubated with the primary antibodies against Bsep (1:200) and ZO-1 (1:500) (overnight, 4°C), Ntcp (1:200) and Na^+/K^+-ATPase (1:100), or β_1 integrin subunit active conformation (1:100) and $\alpha_5\beta_1$ integrin dimer (1:100), washed thrice, and stained with an anti-mouse-FITC and an anti-rabbit Cy3-conjugated antibody (1:500 in PBS, 2 h, room temperature, respectively). To visualize filamentous actin in order to document cell shape, FITC-coupled phalloidin (1 µg/ml) was applied. Following immunofluorescence staining, samples were covered with Fluoromount-G reagent and visualized by confocal laser scanning microscopy (CLSM) using LSM510 META (Zeiss, Oberkochen, Germany). Average pixel intensities for the red channel were calculated in

ImageJ by separating the red channel from each image and extracting the data with the Histogram function.

Densitometric fluorescence intensity analysis

Cryosections of perfused rat liver for the analysis at the canalicular membrane were stained for Bsep and for the tight junction protein ZO-1, which forms the sealing border between canalicular and sinusoidal membrane. Apparent integrity and comparability of the canaliculi was assumed when the bordering tight junction lines (detected by the immunostained ZO-1) were intact and run in parallel. Cryosections of perfused rat liver for the analysis at the basolateral membrane were stained for Ntcp and for Na^+/K^+-ATPase (plasma membrane marker protein). Na^+/K^+-ATPase profiles were selected according apparent integrity and comparability. Acceptable Na^+/K^+-ATPase intensity profiles have a sufficiently high peak fluorescence in the central part (corresponding to the basolateral membrane) and low intracellular fluorescence. For negative controls, primary antibodies were omitted in each experiment. Densitometric analysis was performed as described previously[15,16]. For densitometric analysis using digitalized microscopic pictures of the membranes, the software Image-Pro Plus (Media Cybernetics, Rockville, USA) was used. The profile of the fluorescence intensity was measured over a thick line at a right angle to the membrane. The length of the line was always 8 µm. The mean fluorescence intensity to each pixel over the line perpendicular to the length was calculated by Image-Pro Plus. Each measurement was normalized to the sum of all intensities of the respective measurement. Values are given as means ± SEM. Densitometric analysis of protein distribution in immunofluorescence images were performed using Wilcoxon rank sum test. $p < 0.05$ was considered statistically significant. Data from at least 10 different areas per tissue sample and from at least three independent liver preparations were processed.

Immunoblot analysis

Liver samples were immediately lysed at 4°C by using a lysis buffer containing 20 mmol/l Tris-HCl (pH 7.4), 140 mmol/l NaCl, 10 mmol/l NaF, 10 mmol/l sodium pyrophosphate, 1% (*v/v*) Triton X-100, 1 mmol/l EDTA, 1 mmol/l EGTA, 1 mmol/l sodium vanadate, 20 mmol/l β-glycerophosphate, protease inhibitor (cOmplete™, Roche Diagnostics, Mannheim, Germany) and phosphatase inhibitor cocktail (PhosSTOP™, Roche Diagnostics, Mannheim, Germany). The

lysates were kept on ice for 10 min and then centrifuged at 8000 rpm for 8 min at 4°C, and aliquots of the supernatant were taken for protein determination using the Bio-Rad protein assay (Bio-Rad Laboratories, Munich, Germany). Equal amounts of protein were subjected to sodium dodecyl sulfate / polyacrylamide gel electrophoresis and transferred onto nitrocellulose membranes using a semidry transfer apparatus (GE Healthcare, Freiburg, Germany). Membranes were blocked for 60 min in 5% (*w/v*) bovine serum albumin or 5% (*w/v*) milk powder containing 20 mmol/l Tris (pH 7.5), 150 mmol/l NaCl, and 0.1% Tween 20 (TBS-T) and exposed to primary antibodies overnight at 4°C. After washing with TBS-T and incubation at room temperature for 2 h with horseradish peroxidase-coupled anti-mouse or anti-rabbit IgG antibody, respectively (all diluted 1:10,000), the immunoblots were washed extensively, and bands were visualized using the ChemiDoc^{TM} Touch Imaging System from Bio-Rad (Munich, Germany). Semi-quantitative evaluation was carried out by densitometry using the Image Lab Touch Software from Bio-Rad. Protein phosphorylation is given as the ratio of detected phospho-protein/total protein.

Immunoprecipitation

Liver samples were harvested in lysis buffer containing 136 mmol/l NaCl, 20 mmol/l Tris-HCl, 10% (*v/v*) glycerol, 2 mmol/l EDTA, 50 mmol/l β-glycerophosphate, 20 mmol/l sodium pyrophosphate, 0.2 mmol/l Pefablock, 5 mg/l aprotinin, 5 mg/l leupeptin, 4 mmol/l benzamidine, 1 mmol/l sodium vanadate, supplemented with 1% (*v/v*) Triton X-100. The protein amount was determined as described above. Samples containing equal protein amounts were incubated for 2 h at 4°C with the respective antibody in order to immunoprecipitate the required protein. Then protein A-/G-agarose (Santa Cruz Biotechnology, Heidelberg, Germany) was added and incubated at 4°C overnight. Immunoprecipitates were washed thrice with lysis buffer supplemented with 0.1% (*v/v*) Triton X-100 and then transferred to Western blot analysis as described above.

Supplementary Figures

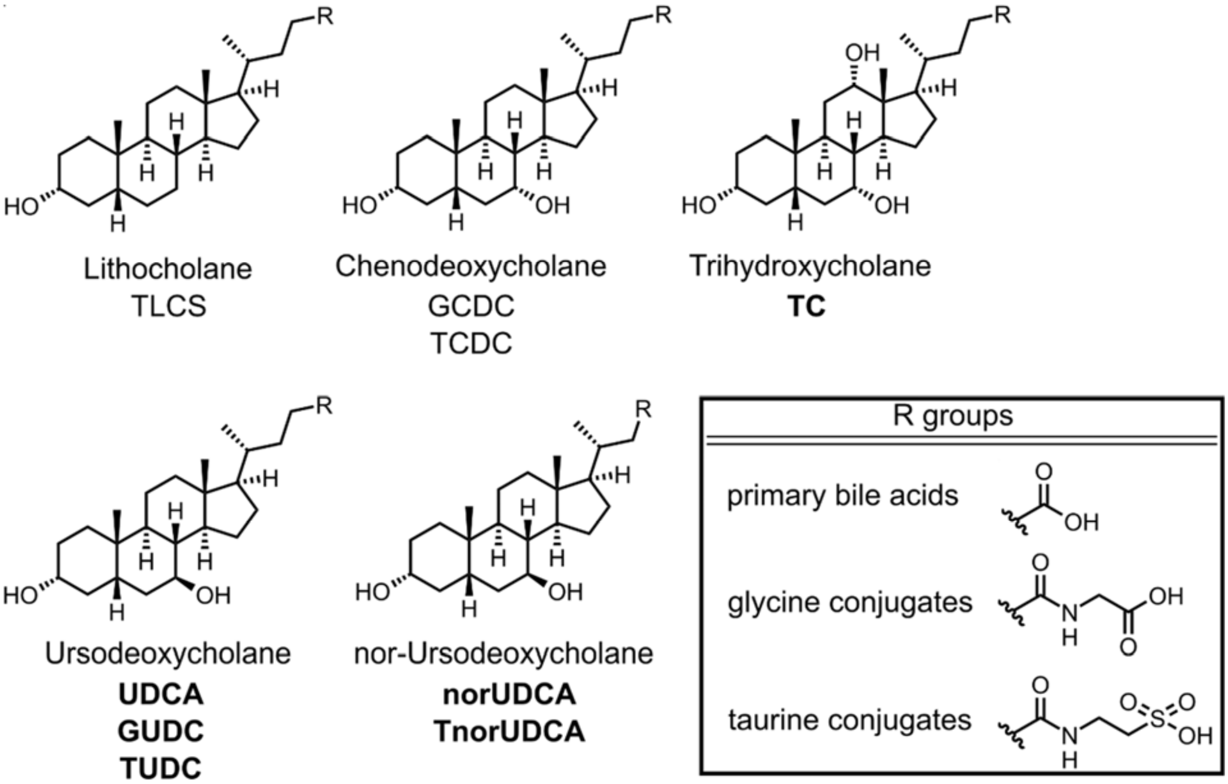

Supplementary Figure 1. Primary and conjugated bile acids. Chemical structures of primary and conjugated bile acids. Bile acids for which agonistic activity towards $\alpha_5\beta_1$ integrins is known or has been determined in this study and bile acids for which antagonistic activity is known are marked in bold.

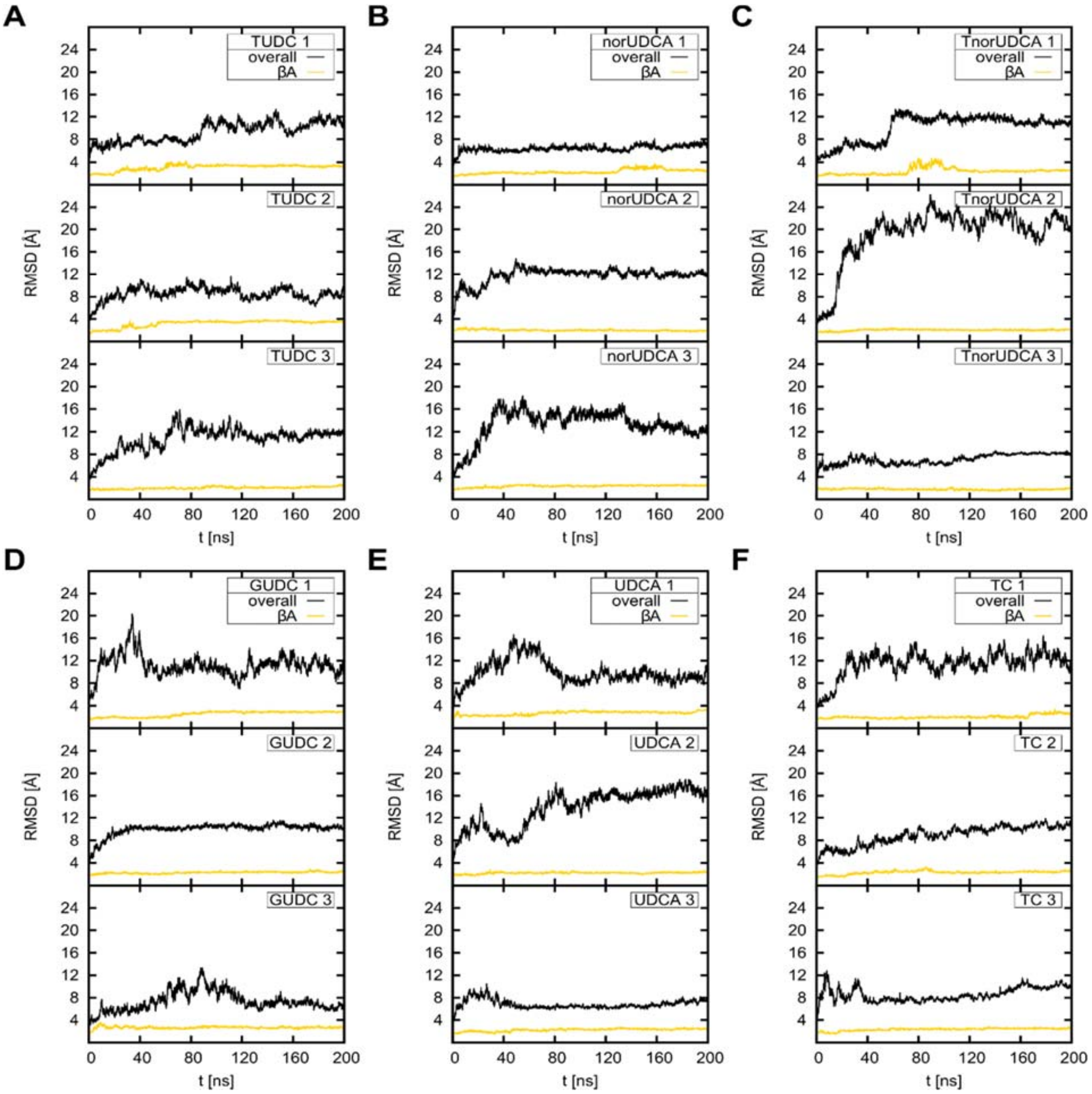

Supplementary Figure 2. Structural stability during the MD simulations. Root mean square deviation (RMSD) of the overall protein structure (black lines) and the βA domain (yellow lines) during three (rows) MD simulations of the complexes between $\alpha_5\beta_1$ integrin and (A) TUDC, (B) *nor*UDCA, (C) T*nor*UDCA, (D) GUDC, (E) UDCA, and (F) TC.

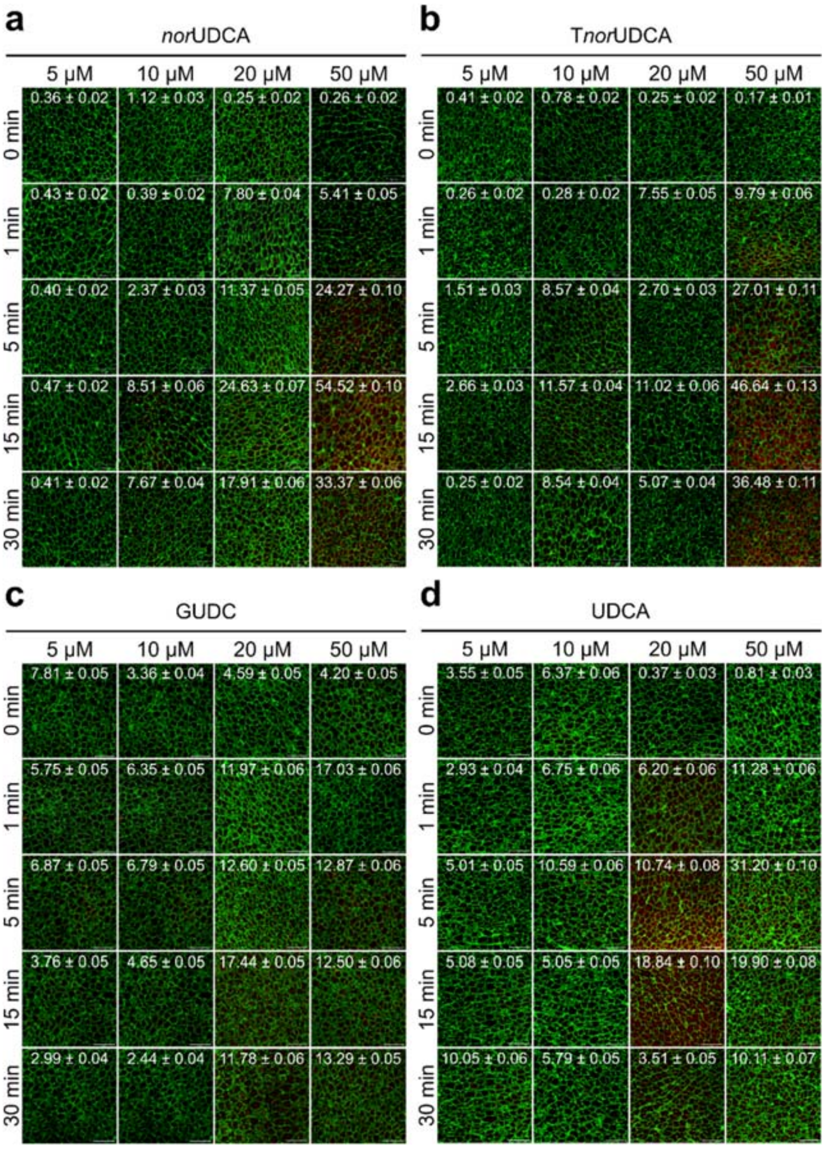

Supplementary Figure 3. Effects of norUDCA, TnorUDCA, GUDC and UDCA on β1 integrin activation. Rat livers were perfused with (a) norUDCA, (b) TnorUDCA, (c) GUDC, and (d) UDCA for up to 60 min with the concentrations indicated. Liver samples were immunostained for the active conformation of β_1 integrin (red) and Na^+-K^+-ATPase (green).

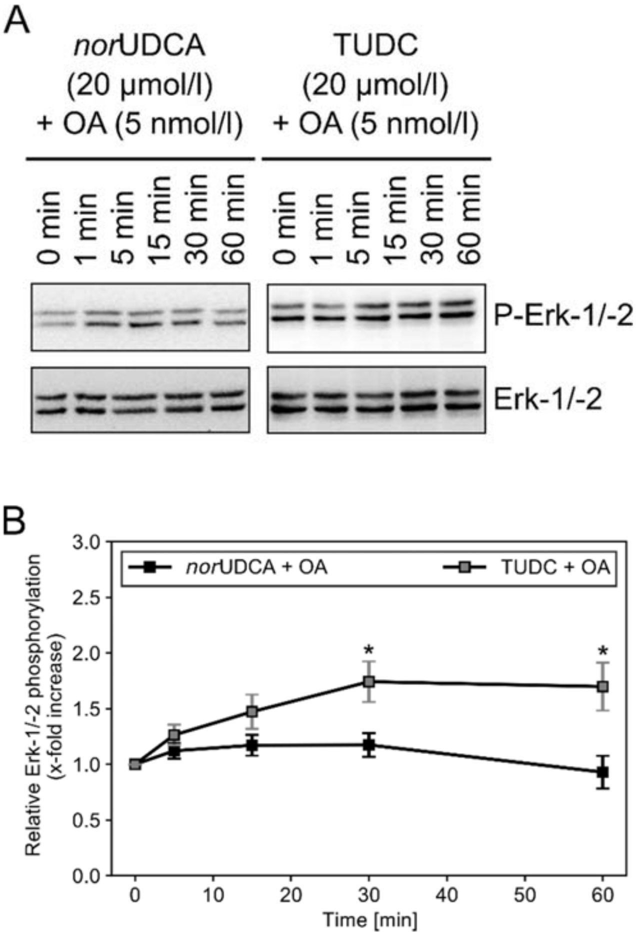

Supplementary Figure 4. Effects of okadaic acid on *nor*UDCA- and TUDC-induced Erk-1/-2 activation. Rat livers were perfused with *nor*UDCA or TUDC (20 µmol/l each) in the presence of okadaic acid (OA) for up to 60 min. Liver samples were taken at the time points indicated. Phosphorylation of Erk-1/-2 was analyzed by (A) Western blot using specific antibodies and (B) subsequent densitometric analysis. Levels of phosphorylated Erk-1/-2 were normalized to total Erk-1/-2. Phosphorylation at $t = 0$ was arbitrarily set to 1. Data represent the mean (mean ± SEM) of at least three independent experiments; *, Statistically significant compared to the unstimulated control ($t = 0$) (one-way ANOVA, Dunnett´s *post hoc* test).

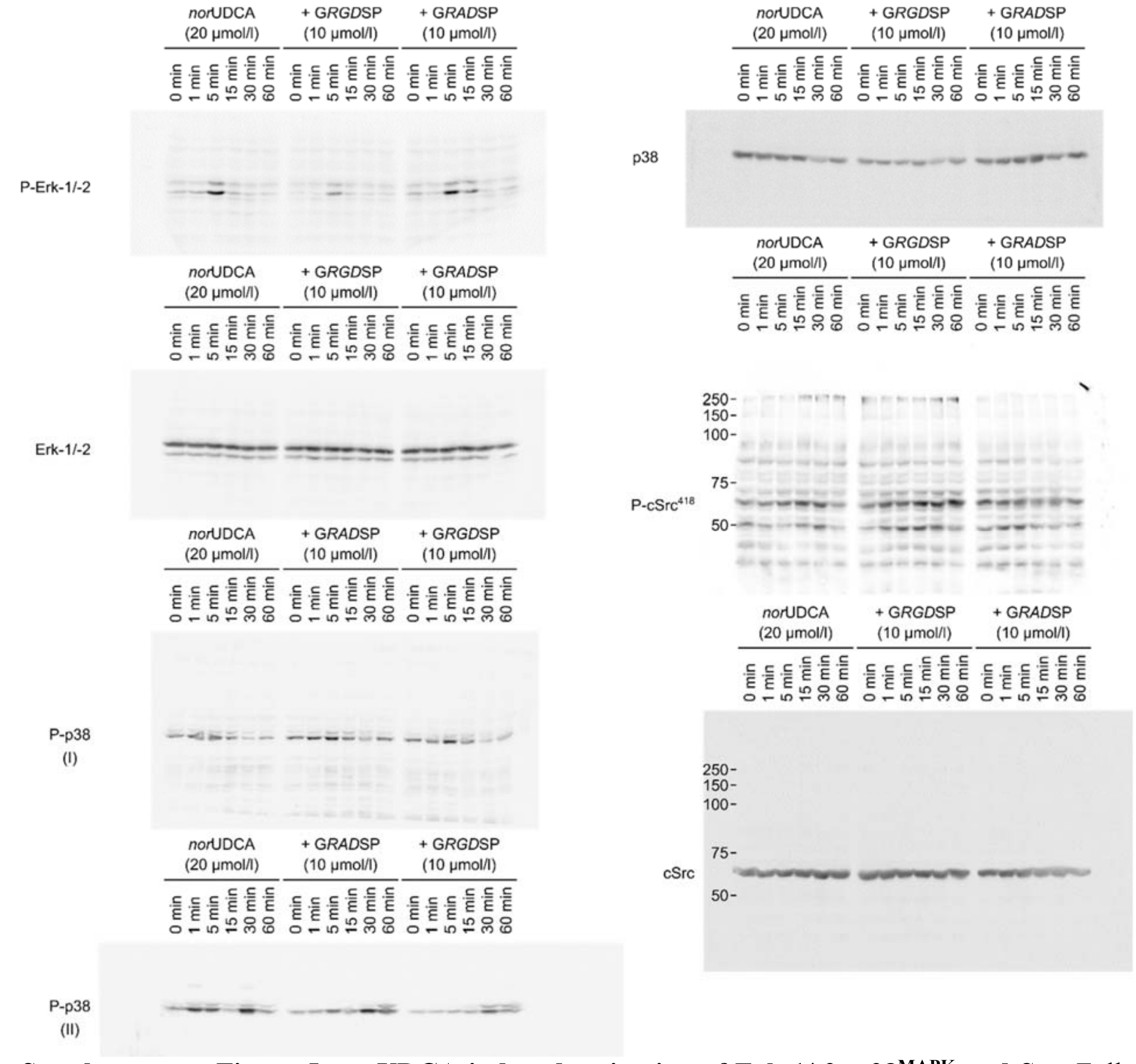

Supplementary Figure 5. *nor*UDCA-induced activation of Erk-1/-2, p38^{MAPK} and Src. Full-length blots of cropped blots from Figure 5a in the main text.

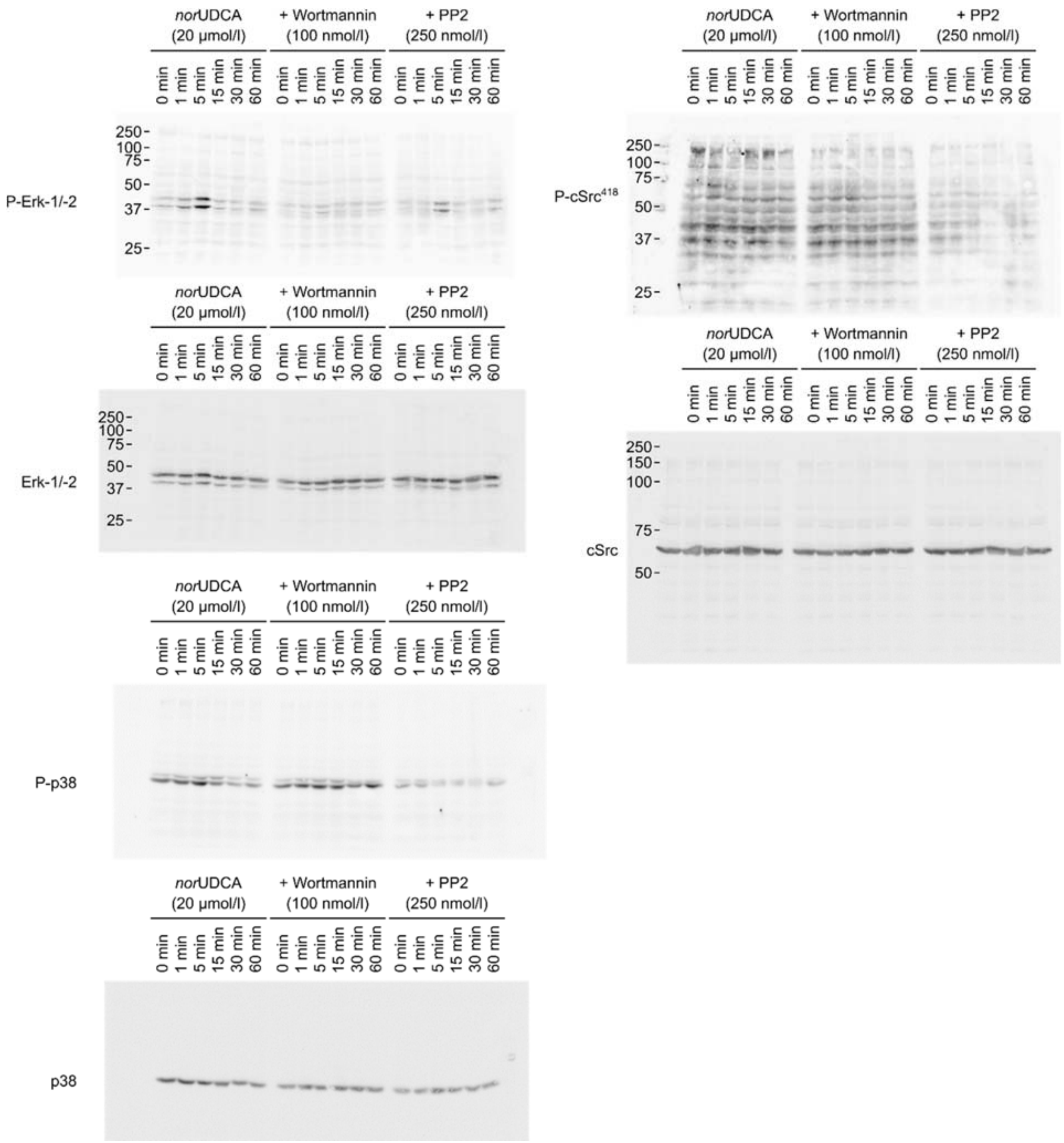

Supplementary Figure 6. *nor*UDCA-induced activation of Erk-1/-2, p38^{MAPK} and Src. Full-length blots of cropped blots from Figure 5b in the main text.

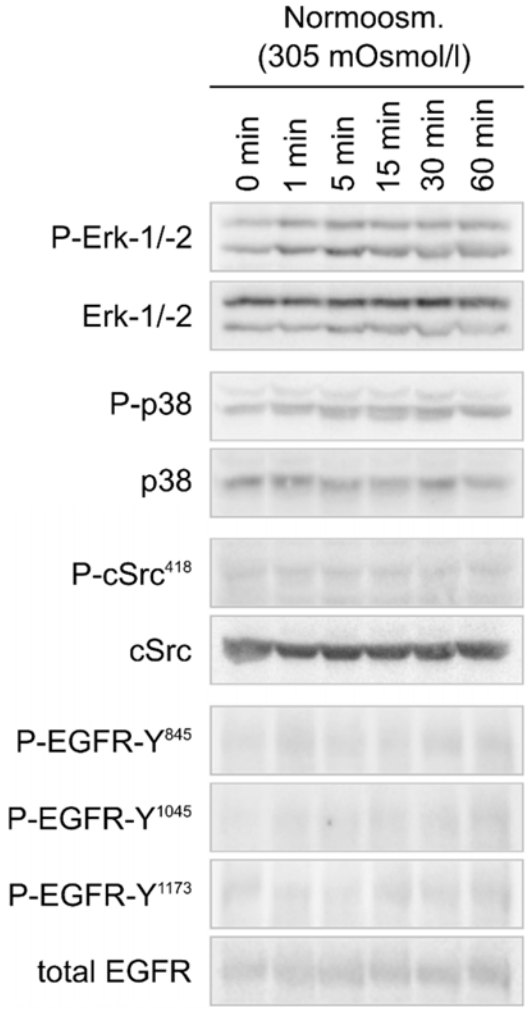

Supplementary Figure 7. Control perfusion experiments. Rat livers were perfused with control buffer for up to 60 min. Liver samples were taken at the time points indicated. Activation of Erk-1/-2, $p38^{MAPK}$, cSrc, EGFR Tyr^{845}, Tyr^{1045}, and Tyr^{1173} were analyzed by Western blot using specific antibodies. Total Erk-1/-2, $p38^{MAPK}$, cSrc, and EGFR served as respective loading controls. Representative pictures of at least three independent experiments are depicted. Blots were cropped to focus on the area of interest. No effect on the phosphorylation of kinases and the EGFR were observed under control conditions.

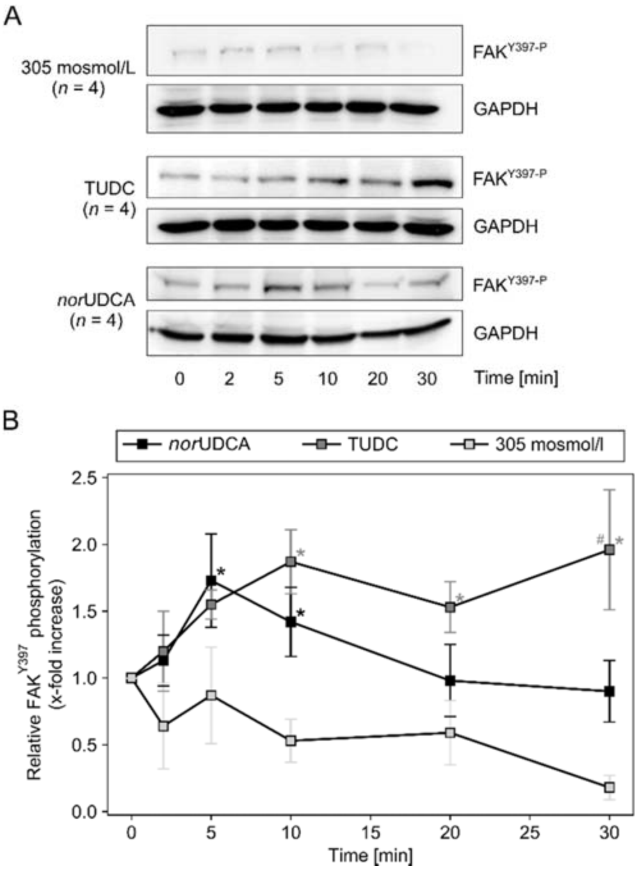

Supplementary Figure 8. Phosphorylation of FAK^{Y397} in *nor*UDCA- and TUDC-perfused rat livers. The livers were perfused either with normoosmotic medium (305 mosmol/l) or with TUDC (20 µmol/l) and *nor*UDCA (20 µmol/l), and the protein samples were gathered at the indicated time points (0-30 min). Thereafter, phosphorylation of FAK^{Y397} was analyzed by Western blotting using specific antibodies. Glyceraldehyde 3-phosphate dehydrogenase (GAPDH) served as a loading control. Blots were cropped to focus on the area of interest. FAK^{Y397} phosphorylation was significantly increased in the TUDC-perfused livers within 10 min and lasted for up to 30 min compared to livers perfused with normoosmotic medium. However, FAK^{Y397} phosphorylation was not significantly increased in *nor*UDCA perfused livers compared to controls. (A) Representative blots are shown from four independent experiments. (B) Phosphorylation of focal adhesion kinase (FAK^{Y397}) was analyzed densitometrically, and the means of measurements in each of the four individual experiments for each condition are shown (means ± SEM). *: Statistically significant against “305 mosmol/l” ($p < 0.05$, $n = 4$, two-way ANOVA, Bonferroni *post hoc* test).

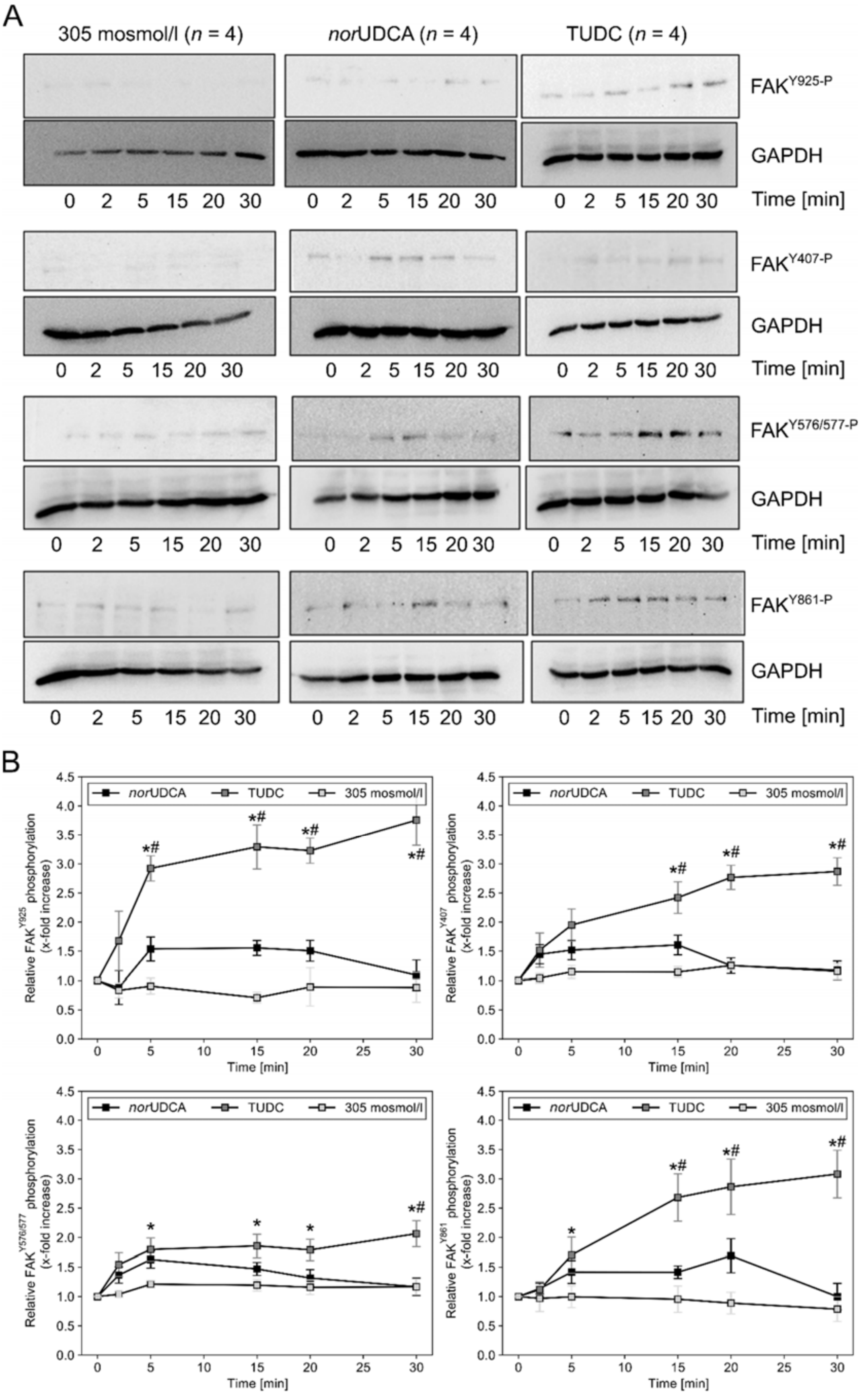
A
305 mosmol/l (n = 4)
norUDCA (n = 4)
TUDC (n = 4)
FAK^Y925-P
GAPDH
0 2 5 15 20 30
Time [min]
FAK^Y407-P
GAPDH
Time [min]
FAK^Y576/577-P
GAPDH
Time [min]
FAK^Y861-P
GAPDH
Time [min]
B
norUDCA
TUDC
305 mosmol/l
Relative FAK^Y925 phosphorylation (x-fold increase)
Relative FAK^Y407 phosphorylation (x-fold increase)
Relative FAK^Y576/577 phosphorylation (x-fold increase)
Relative FAK^Y861 phosphorylation (x-fold increase)
Time [min]
*#
*

Supplementary Figure 9. Phosphorylation of FAK$^{Y925,861,576/577,407}$ in *nor*UDCA- and TUDC-perfused rat livers. The livers were perfused either with normoosmotic medium (305 mosmol/l) or with TUDC (20 µmol/l) and *nor*UDCA (20 µmol/l), and the protein samples were gathered at the indicated time points (0-30 min). Thereafter, phosphorylation of FAK^{Y925}, FAK^{Y407}, FAK$^{Y576/577}$, and FAK^{Y861} was analyzed by Western blotting using specific antibodies. Glyceraldehyde 3-phosphate dehydrogenase (GAPDH) served as a loading control. Blots were cropped to focus on the area of interest. FAK$^{Y925,861,576/577,407}$ phosphorylation was significantly increased in the TUDC-perfused livers within 5-15 min and lasted for up to 30 min compared to livers perfused with normoosmotic medium. However, phosphorylation was not significantly increased in *nor*UDCA perfused livers compared to controls. (A) Representative blots are shown from four independent experiments. (B) Phosphorylation of focal adhesion kinase (FAK$^{Y925,861,576/577,407}$) was analyzed densitometrically, and the means of measurements in each of the four individual experiments for each different condition are shown (means ± SEM). *: Statistically significant against "305 mosmol/l"; #: Statistically significant against "*nor*UDCA" ($p < 0.05$, $n = 4$, two-way ANOVA, Bonferroni *post hoc* test).

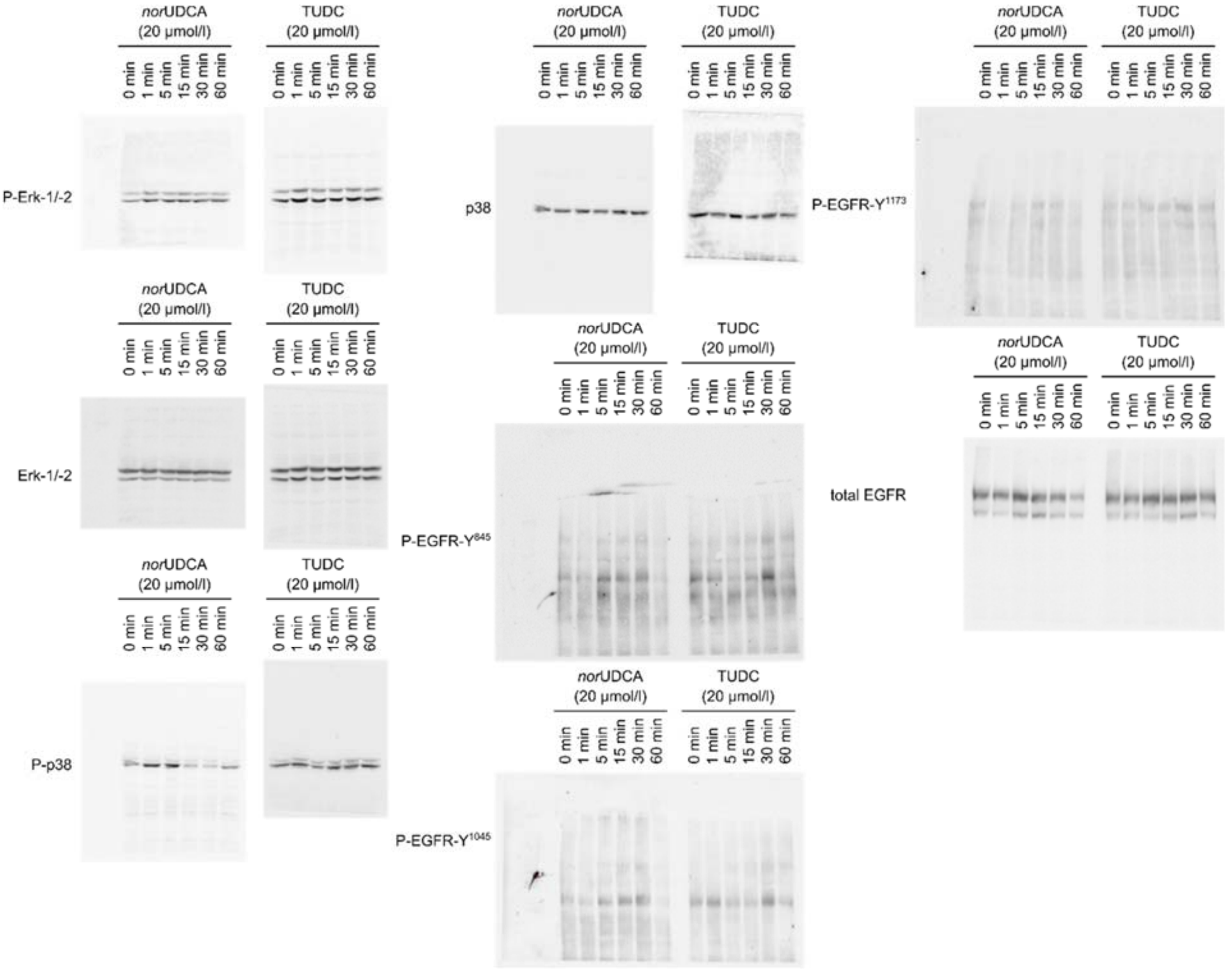

Supplementary Figure 10. Comparison between *nor*UDCA- and TUDC-induced Erk-1/-2, p38^{MAPK} and EGFR activation. Full-length blots of cropped blots from Figure 6a in the main text.

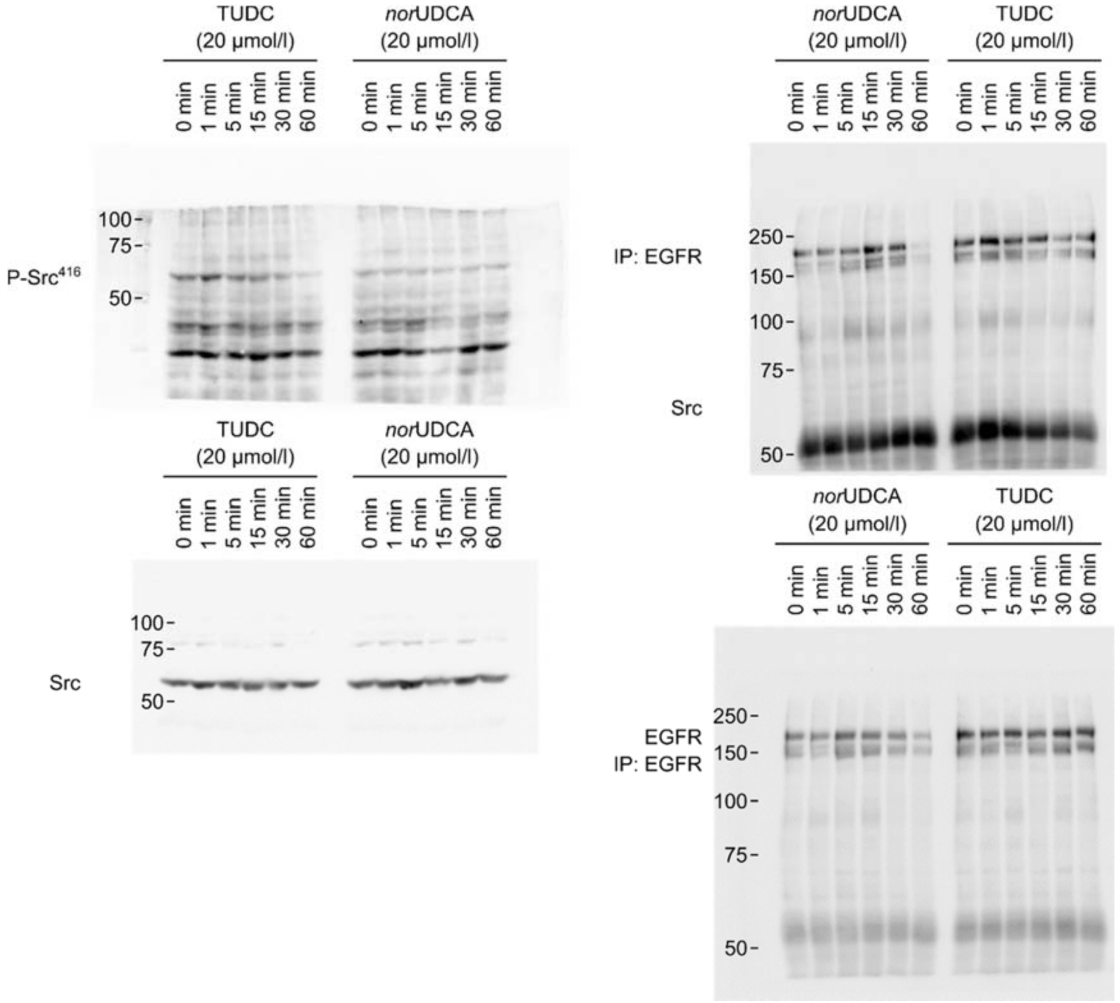

Supplementary Figure 11. Comparison between *nor*UDCA- and TUDC-induced c-Src activation and EGFR/c-Src association. Full-length blots of cropped blots from Figure 7a in the main text.

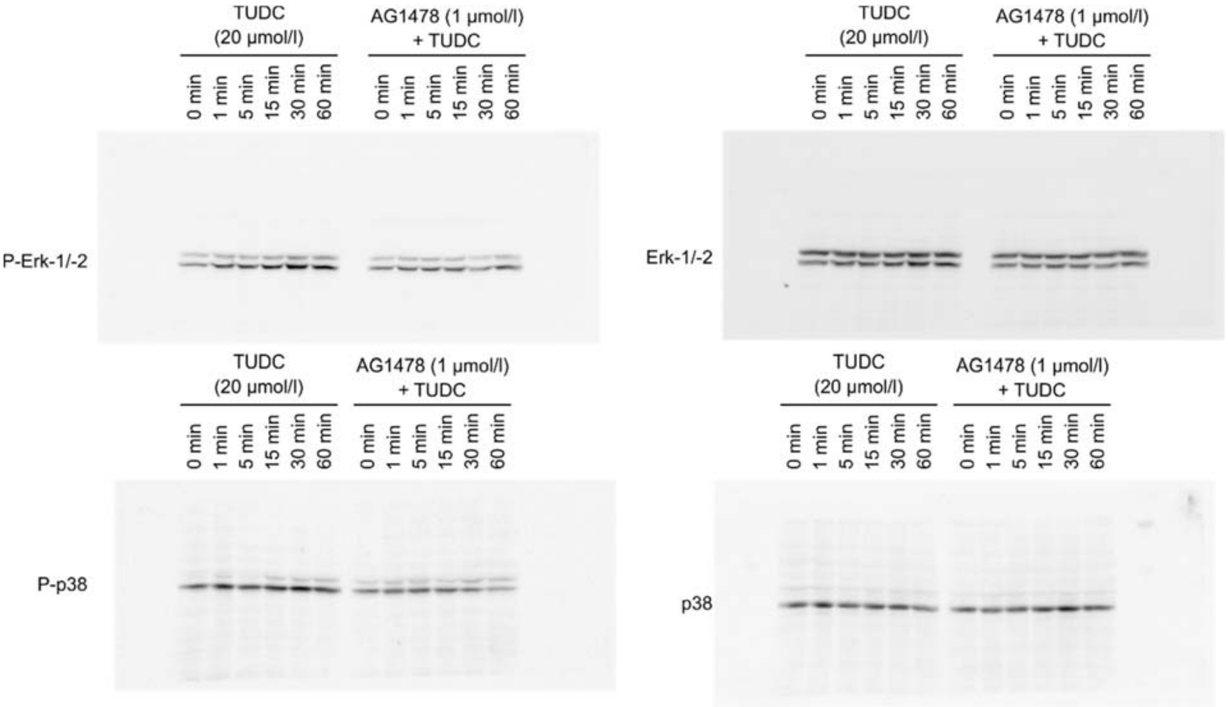

Supplementary Figure 12. TUDC-induced dual activation of Erk-1/-2 and p38^{MAPK} and Bsep insertion into the canalicular membrane are dependent on EGFR phosphorylation. Full-length blots of cropped blots from Figure 8a in the main text.

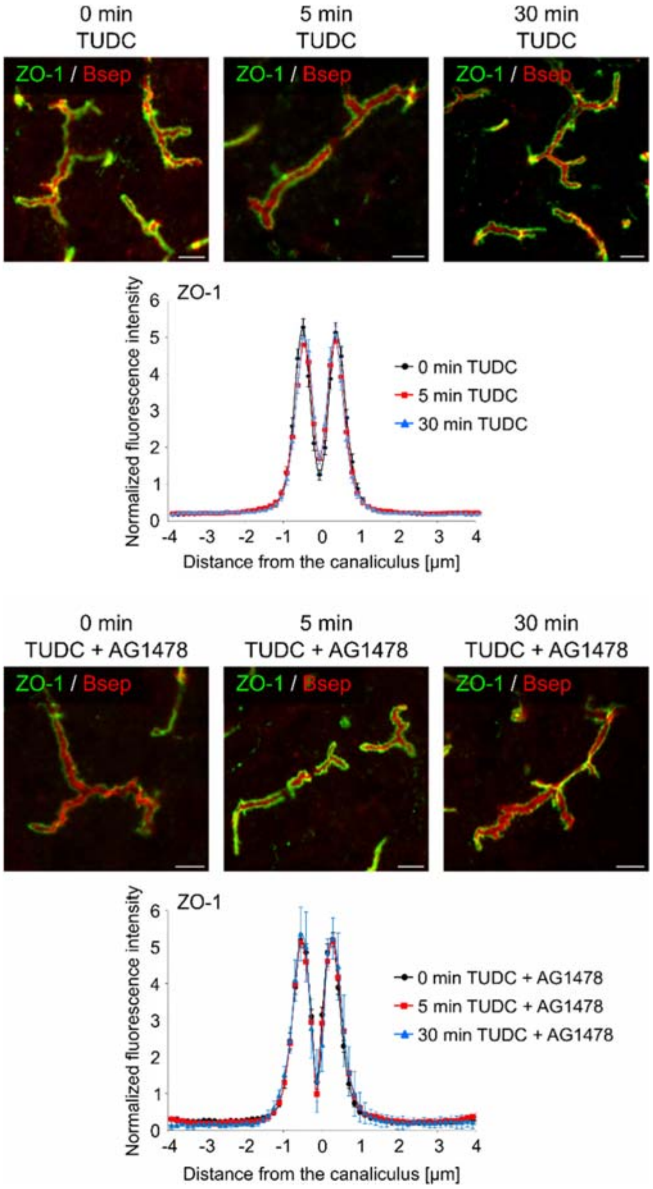

Supplementary Figure 13. Immunohistochemical determination of Bsep and ZO-1 distribution in TUDC perfused rat livers. For experimental details see caption of Fig. 8. Cryosections from perfused rat liver were immunostained for Bsep and ZO-1, fluorescence images were recorded by confocal LSM, and analyzed densitometrically. Representative pictures of at least three independent experiments are depicted. The scale bar corresponds to 5 µm. TUDC led to insertion of intracellular Bsep into the canalicular membrane, which was inhibited by AG1478 (see also Fig. 8). Liver perfusion experiments with *nor*UDCA resulted in no significant changes of ZO-1 fluorescence profiles. Means ± SEM of 30 measurements in each of at least three individual experiments for each condition are shown.

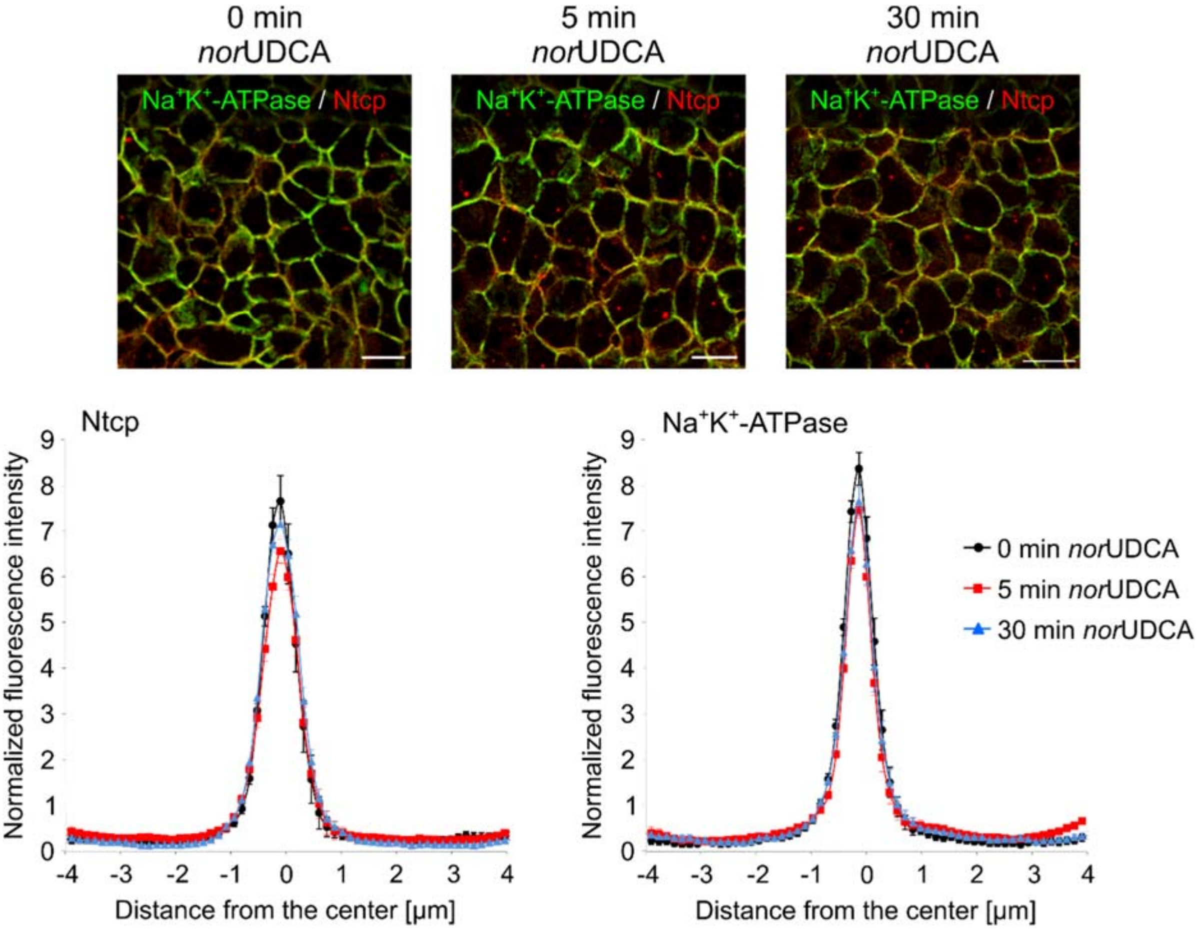

Supplementary Figure 14. Regulation of Ntcp in *nor*UDCA-perfused rat liver. Rat livers were perfused with *nor*UDCA (20 µmol/l) for up to 60 min, immunostained for Ntcp and Na^+/K^+-ATPase. The densitometric analysis of fluorescence profiles and intensity of Ntcp and Na^+/K^+-ATPase distribution are shown. Representative pictures of at least three independent experiments are depicted. The scale bars correspond to 10 µm. Under control conditions (black, $t = 0$ min), Ntcp-bound fluorescence was largely localized in the center of the basolateral membrane. No significant changes of Ntcp and Na^+/K^+-ATPase fluorescence profiles were observed after perfusion with *nor*UDCA (blue, $t = 5$ min; red, $t = 30$ min). Means ± SEM of 30 measurements in each of at least three individual experiments for each condition are shown.

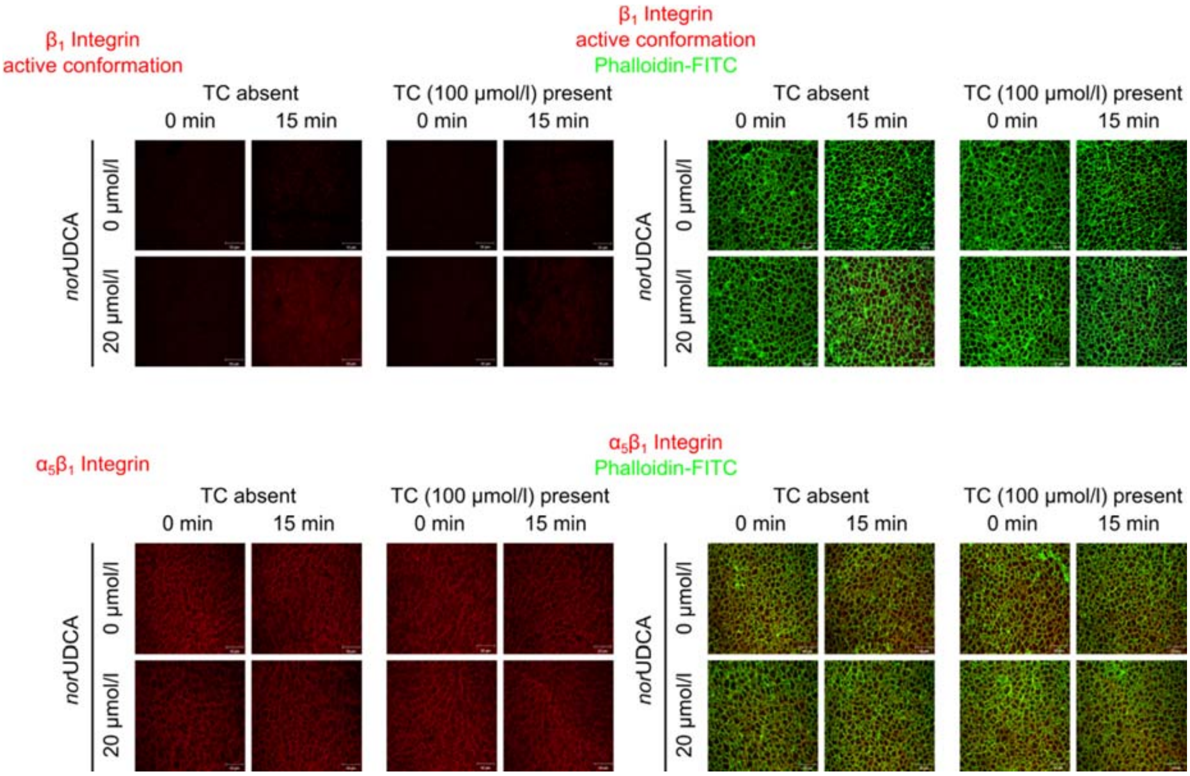

Supplementary Figure 15. Inhibition of *nor*UDCA-induced β_1 integrin activation by TC. Rat livers were perfused with *nor*UDCA (20 µmol/l) for 15 min. Experiments were carried out in the absence or presence of TC (100 µmol/l), which was added 30 min prior to *nor*UDCA addition. Liver samples were taken at the time points indicated. Cryosections were stained for the β_1 integrin subunit or for total $\alpha_5\beta_1$ integrin, and fluorescence images were recorded by LSM. In order to visualize cell shapes and integrity of the cytoskeleton, filamentous actin was labeled by use of FITC-coupled phalloidin as a control. The scale bar corresponds to 50 µm. In the absence of TC, *nor*UDCA induced within 15 min an activation of the β_1 integrin subunit, whereas in the presence of TC the effect was abolished. Immunoreactivity of the total $\alpha_5\beta_1$ integrin heterodimer as well as the integrity of the cytoskeleton remained unchanged under these conditions.

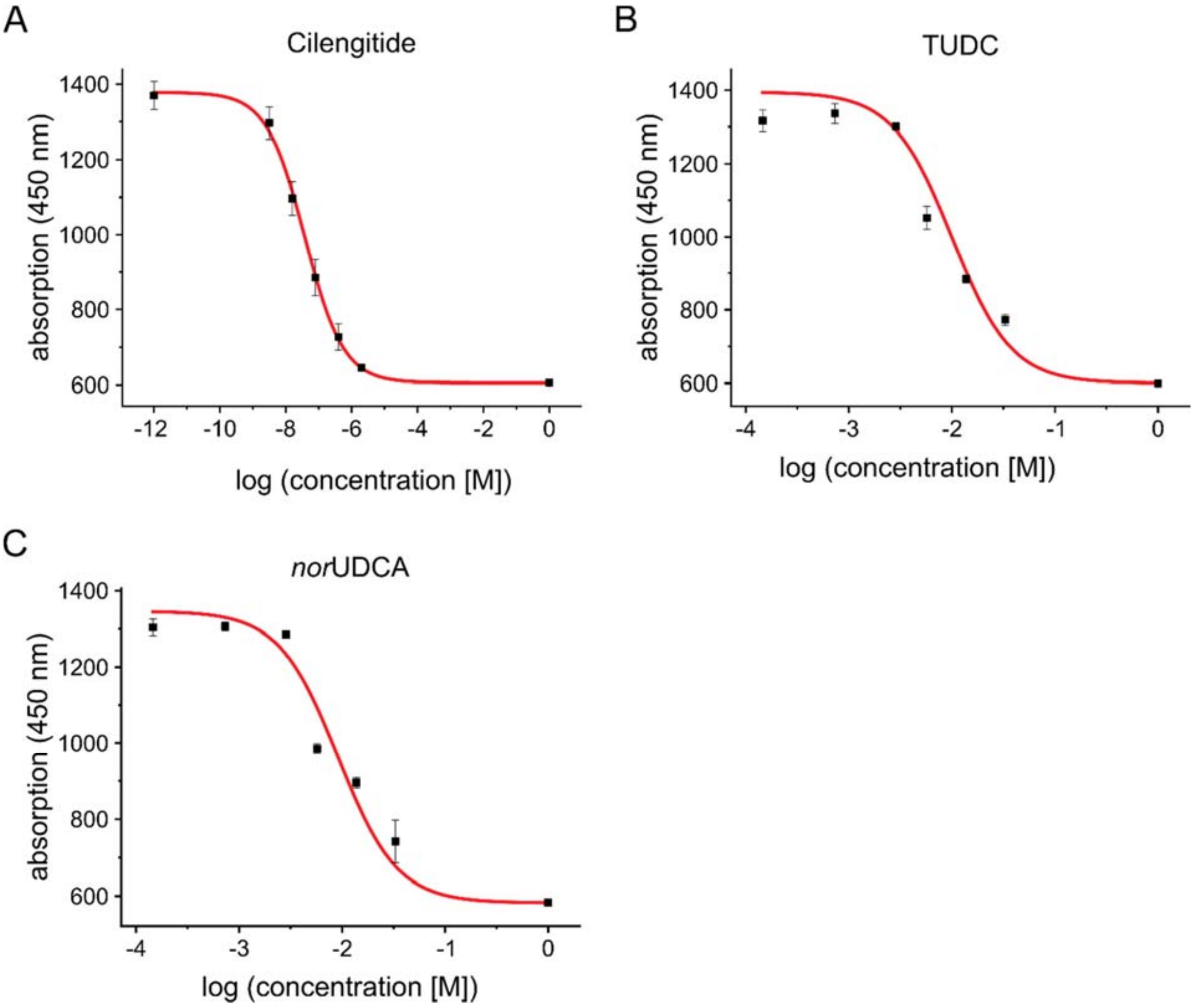

Supplementary Figure 16. Binding of TUDC and *nor*UDCA towards $\alpha_5\beta_1$. Absorption-concentration curves obtained from the competitive ELISA-based assay for (A) Cilengitide (reference compound), (B) TUDC, and (C) *nor*UDCA binding to $\alpha_5\beta_1$ integrin. The red curves represent a sigmoidal fit to 32 data points, obtained from two serial dilution rows.

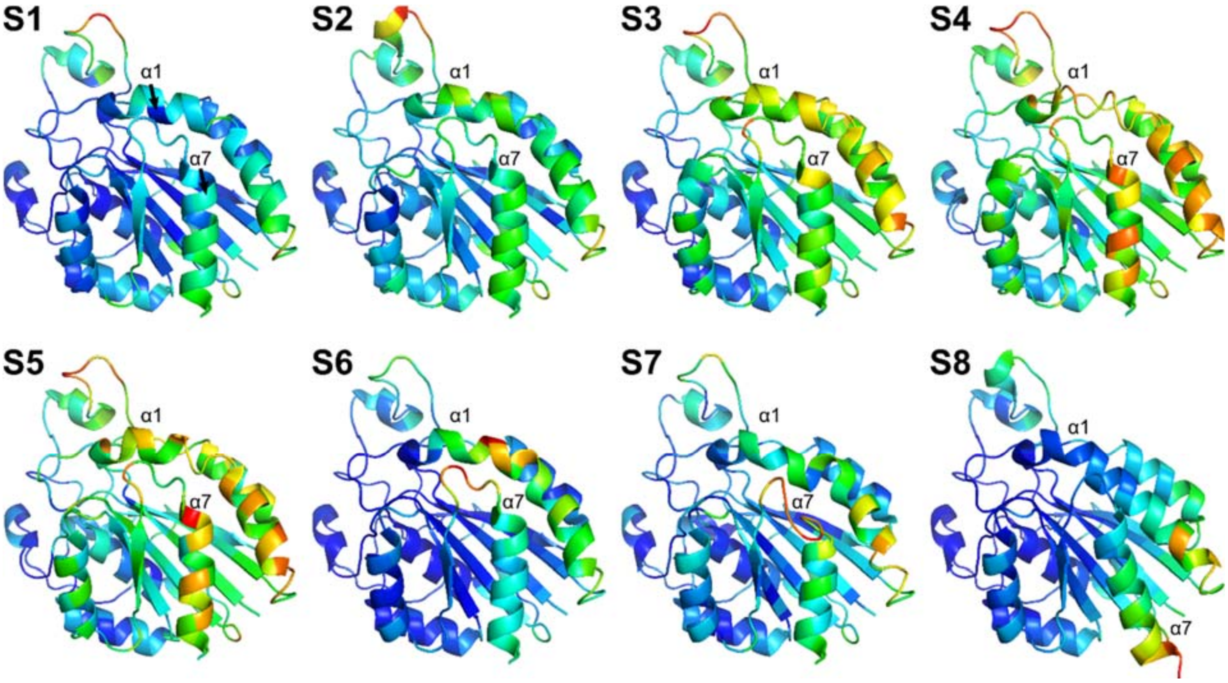

Supplementary Figure 17. Flexibility changes in the βA domain upon activation. Structural time course (S1 → S8) of integrin activation in the βA domain of $\alpha_{IIb}\beta_3$ integrins derived from crystal structures (PDB entries: 3ZDX, 3ZDY, 3ZDZ, 3ZE0, 3ZE1, 3ZE2)[17]. Colors denote the average B factor of the amino acids, ranging from 0 (blue) to the maximum in the βA domain of the respective structure (red).

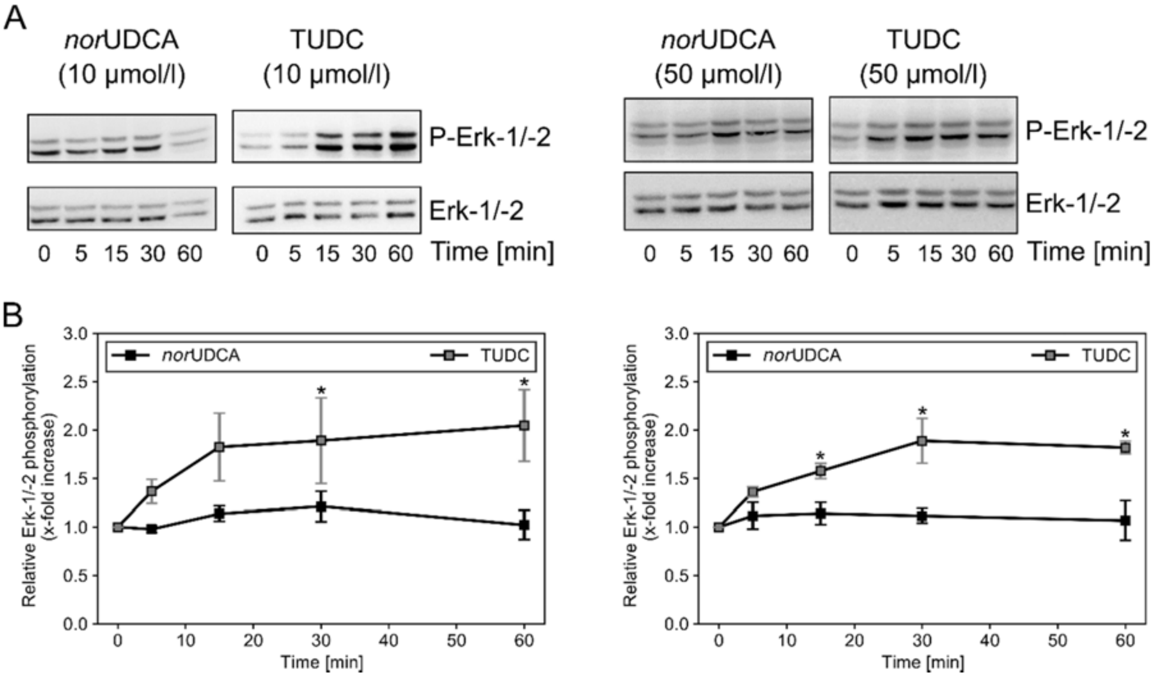

Supplementary Figure 18. Comparison between *nor*UDCA- and TUDC-induced Erk-1/-2 activation. Rat livers were perfused with *nor*UDCA or TUDC (10 or 50 µmol/l each) for up to 60 min. Liver samples were taken at the time points indicated. Phosphorylation of Erk-1/-2 was analyzed by (A) Western blot using specific antibodies and (B) subsequent densitometric analysis. Levels of phosphorylated Erk-1/-2 were normalized to total Erk-1/-2. Phosphorylation at $t = 0$ was arbitrarily set to 1. Data represent the mean (mean ± SEM) of at least three independent experiments; *: Statistically significant compared to the unstimulated control ($t = 0$) (one-way ANOVA, Dunnett´s *post hoc* test).

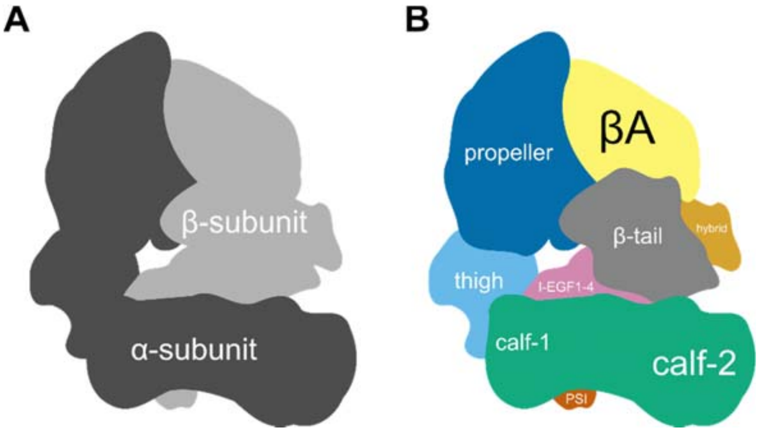

Supplementary Figure 19. Subunit (A) and domain organization (B) of the $\alpha_5\beta_1$ integrin ectodomain. (A) Illustration of the subunit organization in $\alpha_5\beta_1$ integrin. The α-subunit is depicted in dark gray, the β-subunit is depicted in light gray. (B) Illustration of the domain organization in $\alpha_5\beta_1$ integrin. β-Propeller domain: dark blue, thigh domain: light blue, calf-1/2 domains: green; βA domain: yellow, hybrid domain: ochre, PSI domain: dark orange, I-EGF 1-4 domains: pink, β-tail domain: grey.

Supplementary Tables

Supplementary Table 1. Domain-wise minimum, maximum and average RMSD values.[a]

α-subunit RMSD		propeller min	propeller mean	propeller max	thigh min	thigh mean	thigh max	calf-1 min	calf-1 mean	calf-1 max	calf-2 min	calf-2 mean	calf-2 max
TUDC	1	1.53	1.99	2.71	1.87	2.47	3.37	1.49	2.04	2.58	2.13	4.51	5.86
	2	1.58	2.08	2.60	1.63	3.05	3.83	1.68	2.21	2.73	2.24	4.02	5.30
	3	1.48	2.14	2.82	1.59	2.60	3.41	1.81	2.31	2.76	2.11	5.13	6.61
*nor*UDCA	1	1.61	2.33	3.18	1.41	2.59	3.51	1.55	2.07	2.53	2.04	4.86	6.04
	2	1.34	2.24	3.02	1.69	2.94	4.04	1.59	2.57	3.19	2.25	3.33	4.58
	3	1.38	2.06	2.56	1.62	2.62	3.37	1.67	2.27	2.82	2.19	4.49	5.25
T*nor*UDCA	1	1.69	2.64	3.28	1.57	2.68	3.18	1.63	2.22	2.74	2.14	4.62	5.36
	2	1.28	2.18	2.74	1.66	2.67	3.54	1.60	2.52	3.08	2.07	4.20	5.16
	3	1.56	1.90	2.43	1.48	2.46	3.38	1.56	2.16	2.87	2.26	3.18	4.25
GUDC	1	1.40	2.17	3.22	1.81	2.42	3.10	1.44	2.21	2.92	2.11	4.76	6.74
	2	1.58	2.51	3.36	1.70	2.82	3.54	1.57	2.48	2.94	2.22	4.32	5.54
	3	1.44	2.02	2.77	1.65	2.38	3.32	1.86	2.47	3.10	2.14	4.44	5.60
UDCA	1	1.55	2.43	3.17	1.73	2.90	3.48	1.54	1.91	2.36	2.36	3.54	4.50
	2	1.42	2.24	2.77	1.75	2.86	3.63	1.71	2.23	2.79	2.12	4.59	5.55
	3	1.42	2.14	3.03	1.65	2.44	3.02	1.56	1.99	2.48	2.38	5.98	6.91
TC	1	1.63	2.42	3.15	1.65	2.58	3.32	1.53	2.17	2.76	2.06	3.37	4.13
	2	1.49	2.26	3.20	1.68	2.53	3.87	1.63	2.29	2.77	2.07	4.16	5.92
	3	1.56	2.10	3.11	1.83	2.74	3.57	1.72	2.78	3.47	2.31	5.58	6.55

β-subunit RMSD		βA min	βA mean	βA max	hybrid min	hybrid mean	hybrid max	PSI min	PSI mean	PSI max	I-EGF3 min	I-EGF3 mean	I-EGF3 max	I-EGF4 min	I-EGF4 mean	I-EGF4 max	β-tail min	β-tail mean	β-tail max
TUDC	1	1.38	3.07	4.23	1.86	3.79	4.50	2.37	5.09	7.16	0.88	1.64	2.64	1.23	3.99	6.02	1.99	4.25	5.59
	2	1.35	3.21	3.89	1.88	3.43	4.53	2.21	5.60	7.26	0.98	2.47	3.35	1.12	1.84	3.25	2.02	4.74	6.16
	3	1.29	2.02	2.66	1.72	3.84	5.06	2.25	5.24	6.77	1.12	3.41	5.86	1.29	2.18	2.86	1.82	4.01	5.27
*nor*UDCA	1	1.26	2.30	3.84	1.69	4.08	4.77	2.34	4.55	6.85	1.00	1.60	2.82	1.17	1.92	2.77	2.14	5.47	6.22
	2	1.33	2.01	2.78	1.85	3.94	4.59	2.56	4.93	6.23	1.15	2.13	3.26	0.96	1.86	2.92	1.85	3.76	4.86
	3	1.37	2.33	2.77	1.79	3.36	4.86	2.95	6.86	8.71	1.03	2.68	4.15	1.18	2.91	4.43	1.83	3.63	4.96
T*nor*UDCA	1	1.40	2.39	4.80	1.50	3.70	4.86	2.09	4.41	6.57	1.01	2.82	5.01	1.19	1.90	2.86	1.86	6.61	7.94
	2	1.39	2.03	2.42	1.55	4.11	4.89	2.37	5.66	7.11	0.97	2.11	3.77	1.03	2.30	3.40	2.10	4.06	5.31
	3	1.37	1.77	2.22	1.92	4.2	5.39	2.90	5.82	7.56	0.99	3.13	5.01	1.09	1.64	2.76	2.10	5.36	8.95
GUDC	1	1.44	2.55	3.17	1.48	3.71	5.01	2.87	4.41	6.00	1.19	3.46	4.93	1.09	2.15	4.01	2.01	3.66	4.92
	2	1.31	2.26	2.89	1.66	3.16	4.66	1.96	5.28	6.75	1.02	2.53	3.38	1.12	1.80	2.44	1.79	3.75	5.13
	3	1.54	2.67	3.65	1.77	3.25	4.55	2.75	5.21	6.73	1.04	2.12	3.19	0.97	1.83	3.15	1.81	3.82	4.94
UDCA	1	1.46	2.71	3.38	1.53	3.70	5.32	2.30	5.99	7.54	0.92	1.65	2.38	1.10	1.78	2.57	1.89	3.69	5.21
	2	1.40	2.20	2.82	1.75	4.26	5.18	2.10	4.06	5.18	1.02	3.44	4.38	1.01	1.84	2.95	1.92	4.54	5.77
	3	1.47	2.25	2.72	1.62	3.41	4.88	2.68	5.20	6.76	1.09	1.84	2.30	1.02	1.62	2.58	1.78	4.47	5.72
TC	1	1.48	2.06	3.36	1.72	4.97	6.89	2.38	4.04	5.39	1.09	2.92	4.44	1.07	1.97	4.01	1.79	4.99	6.94
	2	1.33	2.29	3.28	1.49	4.15	5.40	2.32	4.58	5.91	0.97	2.42	3.09	1.05	1.65	2.41	2.07	3.99	5.47
	3	1.49	2.35	2.78	1.50	3.70	4.74	2.53	5.46	6.81	1.27	2.61	3.78	1.06	1.55	2.63	1.84	4.23	7.11

[a] RMSD values larger than 4 Å and larger than 6 Å are highlighted in orange and red, respectively.

Supplementary Table 2. Angles and distances computed from MD trajectories.[a]

Simulation (number)	α1 kink angle [b]	α7 tilt angle [b]	Propeller-βA distance [c]
TUDC (1)	156.54 ± 0.05	146.48 ± 0.05	37.35 ± 0.004
TUDC (2)	139.98 ± 0.10	130.74 ± 0.08	35.72 ± 0.004
TUDC (3)	145.25 ± 0.08	137.27 ± 0.08	36.97 ± 0.007
TUDC[d]	147.26 ± 0.13	138.16 ± 0.12	36.68 ± 0.010
*nor*UDCA (1)	150.23 ± 0.05	140.53 ± 0.05	35.61 ± 0.002
*nor*UDCA (2)	150.22 ± 0.05	137.21 ± 0.08	36.89 ± 0.005
*nor*UDCA (3)	147.29 ± 0.04	137.44 ± 0.04	35.63 ± 0.003
*nor*UDCA[d]	149.25 ± 0.08	138.39 ± 0.10	36.04 ± 0.006
T*nor*UDCA (1)	141.21 ± 0.05	130.77 ± 0.05	35.36 ± 0.003
T*nor*UDCA (2)	146.04 ± 0.06	137.48 ± 0.05	36.24 ± 0.003
T*nor*UDCA (3)	141.17 ± 0.04	141.50 ± 0.05	35.07 ± 0.004
T*nor*UDCA[d]	142.81 ± 0.09	136.58 ± 0.09	35.56 ± 0.006
GUDC (1)	142.60 ± 0.05	131.86 ± 0.06	35.27 ± 0.005
GUDC (2)	145.54 ± 0.05	134.80 ± 0.05	36.31 ± 0.003
GUDC (3)	144.91 ± 0.05	142.76 ± 0.06	35.38 ± 0.002
GUDC[d]	144.35 ± 0.09	136.47 ± 0.10	35.65 ± 0.006
UDCA (1)	145.98 ± 0.05	140.64 ± 0.09	36.07 ± 0.006
UDCA (2)	136.43 ± 0.06	127.92 ± 0.05	35.43 ± 0.003
UDCA (3)	136.31 ± 0.07	120.08 ± 0.07	35.90 ± 0.006
UDCA[d]	139.58 ± 0.11	129.55 ± 0.12	35.80 ± 0.006
TC (1)	142.46 ± 0.05	126.11 ± 0.07	35.76 ± 0.004
TC (2)	140.49 ± 0.06	127.91 ± 0.07	35.74 ± 0.005
TC (3)	142.96 ± 0.07	125.81 ± 0.07	35.80 ± 0.007
TC[d, e]	141.97 ± 0.10	126.61 ± 0.12	35.77 ± 0.010
Overall mean[f]	144.20 ± 0.25	134.30 ± 0.27	35.92 ± 0.019

[a] For the geometric parameters, the average over the last 100 ns ± SEM is shown. See Figure 2 in the main text for a definition of these parameters.

[b] In °.

[c] In Å

[d] Data averaged over the three replicate MD simulations performed for each bile acid.

[e] MD simulations with TC served as negative control.

[f] Data averaged over all 18 MD simulations performed for the bile acids.

Supplementary Table 3. Statistical testing for differences in mean values.[a]

α1 tilt angle [b]		TUDC			*nor*UDCA			T*nor*UDCA			GUDC			UDCA			TC		
		1	2	3	1	2	3	1	2	3	1	2	3	1	2	3	1	2	3
TUDC	1																		
TUDC	2	****																	
TUDC	3	****	****																
*nor*UDCA	1	****	****	****															
*nor*UDCA	2	****	****	****	ns														
*nor*UDCA	3	****	****	****	****	****													
T*nor*UDCA	1	****	****	****	****	****	****												
T*nor*UDCA	2	****	****	****	****	****	****	****											
T*nor*UDCA	3	****	****	****	****	****	****	ns	****										
GUDC	1	****	****	****	****	****	****	****	****	****									
GUDC	2	****	****	**	****	****	****	****	****	****	****								
GUDC	3	****	****	****	****	****	****	****	****	****	****	****							
UDCA	1	****	****	****	****	****	****	****	ns	****	****	****	****						
UDCA	2	****	****	****	****	****	****	****	****	****	****	****	****	****					
UDCA	3	****	****	****	****	****	****	****	****	****	****	****	****	****	ns				
TC	1	****	****	****	****	****	****	****	****	****	ns	****	****	****	****	****			
TC	2	****	****	****	****	****	****	****	****	****	****	****	****	****	****	****	****		
TC	3	****	****	****	****	****	****	****	****	****	***	****	****	****	****	****	****	****	
**α7 kink angle [b]**		**TUDC**			***nor*UDCA**			**T*nor*UDCA**			**GUDC**			**UDCA**			**TC**		
		1	2	3	1	2	3	1	2	3	1	2	3	1	2	3	1	2	3
TUDC	1																		
TUDC	2	****																	
TUDC	3	****	****																
*nor*UDCA	1	****	****	****															
*nor*UDCA	2	****	****	****	****														
*nor*UDCA	3	****	****	****	****	****													
T*nor*UDCA	1	****	****	****	****	****	****												
T*nor*UDCA	2	****	****	****	****	****	****	****											
T*nor*UDCA	3	****	****	****	****	****	****	****	****										
GUDC	1	****	****	****	****	****	****	****	****	****									
GUDC	2	****	****	****	****	****	****	****	****	****	****								
GUDC	3	****	****	****	****	****	****	****	****	****	****	****							
UDCA	1	****	ns	****	****	****	***	****	****	****	****	****	****						
UDCA	2	****	****	****	****	****	****	****	****	****	****	****	****	****					
UDCA	3	****	****	****	****	****	****	****	****	****	****	****	****	****	****				
TC	1	****	****	****	****	****	****	****	****	****	****	****	****	****	****	****			
TC	2	****	****	****	****	****	****	****	****	****	****	****	****	****	ns	****	****		
TC	3	****	****	****	****	****	****	****	****	****	****	****	****	****	**	****	****	*	
**Propeller-βA distance [b]**		**TUDC**			***nor*UDCA**			**T*nor*UDCA**			**GUDC**			**UDCA**			**TC**		
		1	2	3	1	2	3	1	2	3	1	2	3	1	2	3	1	2	3
TUDC	1																		
TUDC	2	****																	
TUDC	3	****	****																
*nor*UDCA	1	****	*	****															
*nor*UDCA	2	****	****	****	****														
*nor*UDCA	3	****	****	****	****	****													
T*nor*UDCA	1	****	****	****	****	****	****												
T*nor*UDCA	2	****	***	****	ns	****	****	****											
T*nor*UDCA	3	****	****	****	****	****	****	****	****										
GUDC	1	****	****	****	****	****	****	*	****	****									
GUDC	2	****	****	****	****	****	****	****	****	****	****								
GUDC	3	****	****	****	****	****	****	**	****	****	ns	****							
UDCA	1	****	****	****	****	****	****	****	****	****	****	****	****						
UDCA	2	****	****	****	****	****	****	****	****	****	****	****	****	****					
UDCA	3	****	****	****	****	****	****	**	****	****	****	****	****	****	****				
TC	1	****	****	****	****	****	****	****	****	****	****	****	****	****	****	****			
TC	2	****	****	****	****	****	****	****	****	****	****	****	****	****	****	****	****		
TC	3	****	****	****	****	****	****	****	****	****	****	****	****	****	****	****	****	****	

[a] ****: $p < 0.0001$, ***: $p < 0.001$, **: $p < 0.01$, *: $p < 0.05$, ns: $p \geq 0.05$. Results from redundant tests are omitted.

[b] See Figure 2 in the main text for a definition of the geometric parameters.

Supplementary References

1 Reinehr, R., Gohlke, H., Sommerfeld, A., vom Dahl, S. & Häussinger, D. Activation of Integrins by Urea in Perfused Rat Liver. *J. Biol. Chem.* **285**, 29348-29356, doi:10.1074/jbc.M110.155135 (2010).

2 Gohlke, H., Schmitz, B., Sommerfeld, A., Reinehr, R. & Häussinger, D. α5β1-integrins are sensors for tauroursodeoxycholic acid in hepatocytes. *Hepatology* **57**, 1117-1129, doi:10.1002/hep.25992 (2013).

3 Suite 2012: Maestro, version 9.3 (Schrödinger LLC, New York, NY, 2012).

4 Suite 2012: LigPrep, version 2.5 (Schrödinger LLC, New York, NY, 2012).

5 Shelley, J. C. *et al.* Epik: a software program for pK(a) prediction and protonation state generation for drug-like molecules. *J. Comput.-Aided Mol. Des.* **21**, 681-691, doi:10.1007/s10822-007-9133-z (2007).

6 MacroModel, version 9.9 (Schrödinger LLC, New York, NY, 2012).

7 Gerber, P. R. & Müller, K. MAB, a generally applicable molecular force field for structure modelling in medicinal chemistry. *J. Comput.-Aided Mol. Des.* **9**, 251-268, doi:10.1007/BF00124456 (1995).

8 AMBER 11 (University of California, San Francisco, 2010).

9 Hornak, V. *et al.* Comparison of Multiple Amber Force Fields and Development of Improved Protein Backbone Parameters. *Proteins* **65**, 712-725, doi:10.1002/prot.21123 (2006).

10 Wang, J., Wolf, R. M., Caldwell, J. W., Kollman, P. A. & Case, D. A. Development and Testing of a General Amber Force Field. *J. Comput. Chem.* **25**, 1157-1174, doi:10.1002/jcc.20035 (2004).

11 Åqvist, J. Ion-Water Interaction Potentials Derived from Free-Energy Perturbation Simulations. *J. Phys. Chem.* **94**, 8021-8024, doi:10.1021/j100384a009 (1990).

12 Bayly, C. I., Cieplak, P., Cornell, W. D. & Kollman, P. A. A Well-Behaved Electrostatic Potential Based Method Using Charge Restraints for Deriving Atomic Charges: The RESP Model. *J. Phys. Chem.* **97**, 10269-10280, doi:10.1021/j100142a004 (1993).

13 Jorgensen, W. L., Chandrasekhar, J., Madura, J. D., Impey, R. W. & Klein, M. L. Comparison of simple potential functions for simulating liquid water. *J. Chem. Phys.* **79**, 926-935, doi:10.1063/1.445869 (1983).

14 Darden, T. A., York, D. & Pedersen, L. Particle mesh Ewald - an N·Log(N) method for Ewald sums in large systems. *J. Chem. Phys.* **98**, 10089-10092, doi:10.1063/1.464397 (1993).

15 Schmitt, M., Kubitz, R., Lizun, S., Wettstein, M. & Häussinger, D. Regulation of the Dynamic Localization of the Rat Bsep Gene-Encoded Bile Salt Export Pump by Anisoosmolarity. *Hepatology* **33**, 509-518, doi:10.1053/jhep.2001.22648 (2001).

16 Kubitz, R., D'Urso, D., Keppler, D. & Häussinger, D. Osmodependent Dynamic Localization of the Multidrug Resistance Protein 2 in the Rat Hepatocyte Canalicular Membrane. *Gastroenterology* **113**, 1438-1442, doi:gast.1997.v113.pm9352844 (1997).

17 Zhu, J., Zhu, J. & Springer, T. A. Complete integrin headpiece opening in eight steps. *J. Cell Biol.* **201**, 1053-1068, doi:10.1083/jcb.201212037 (2013).
